# Forging a Functional Cure for HIV: Transcription Regulators and Inhibitors

**DOI:** 10.3390/v14091980

**Published:** 2022-09-07

**Authors:** Sonia Mediouni, Shuang Lyu, Susan M. Schader, Susana T. Valente

**Affiliations:** 1 Department of Immunology and Microbiology, UF Scripps Biomedical Research, 130 Scripps Way, 3C1, Jupiter, FL 33458, USA; 2 Department of Infectious Disease Research, Drug Development Division, Southern Research, 431 Aviation Way, Frederick, MD 21701, USA

**Keywords:** transcription, inhibitors, block-and-lock, latency, functional cure, HIV, epigenetic modulation

## Abstract

Current antiretroviral therapy (ART) increases the survival of HIV-infected individuals, yet it is not curative. The major barrier to finding a definitive cure for HIV is our inability to identify and eliminate long-lived cells containing the dormant provirus, termed viral reservoir. When ART is interrupted, the viral reservoir ensures heterogenous and stochastic HIV viral gene expression, which can reseed infection back to pre-ART levels. While strategies to permanently eradicate the virus have not yet provided significant success, recent work has focused on the management of this residual viral reservoir to effectively limit comorbidities associated with the ongoing viral transcription still observed during suppressive ART, as well as limit the need for daily ART. Our group has been at the forefront of exploring the viability of the block-and-lock remission approach, focused on the long-lasting epigenetic block of viral transcription such that without daily ART, there is no risk of viral rebound, transmission, or progression to AIDS. Numerous studies have reported inhibitors of both viral and host factors required for HIV transcriptional activation. Here, we highlight and review some of the latest HIV transcriptional inhibitor discoveries that may be leveraged for the clinical exploration of block-and-lock and revolutionize the way we treat HIV infections.

## 1. Introduction

Antiretroviral therapy (ART) offers people living with Human Immunodeficiency Virus (HIV, PLWH) a chance to prolong life expectancy by preventing the onset of AIDS and reducing the risk of transmitting HIV to others. The success of ART, however, depends on when the individual begins their prescribed therapy *and* the extent of individual adherence to prescribed doses, dosing intervals and additional medication instructions. An infected individual might experience difficulties in adhering to ART regimens due to cultural beliefs, stigma, cognitive abilities, pill fatigue, comorbidities and/or socioeconomic constraints, including access to adequate healthcare and the ability to afford medications. Long-acting and extended-release antiretroviral (ARV) formulations offer PLWH an alternate approach to once-daily single-tablet dosing. For example, long-acting nano-formulations of cabotegravir and rilpivirine were safe, well-tolerated and efficacious in large, randomized phase 3 studies [1] and approved by the USA Food and Drug Administration (FDA) as a once-month Cabenuva (cabotegravir 200 mg/mL; rilpivirine 300 mg/mL) injections, or every-2-month Cabenuva injections, in those who are virologically suppressed (HIV-1 RNA less than 50 copies per mL). Nonetheless, even these innovative formulations do not obliterate the risk of adherence barriers and/or HIV ARV resistance development. Indeed, the vast majority of PLWH must submit and commit to a lifetime of vigilant disease management using ARVs to prevent AIDS. It follows that a cure for HIV infection is highly desirable and urgently needed.

The persistence of HIV latent reservoirs established early during infection is currently considered the primary obstacle in the HIV cure research and development arena. Generally termed “HIV latency”, clinical HIV latency is defined as the phase of disease whereby HIV is still active but replicates at very low levels. Albeit ARV therapy can reduce viremia and keep HIV from replicating to high levels, virus latency is par for the course for all HIV infections, and, to date, no ARV-based treatments have facilitated a durable cure.

A practical cure for HIV remains elusive despite valiant efforts by the HIV/AIDS research community. Only five instances of PLWH have ever been “cured” and all by means of cell transplants to cure concurrent cancers; the “Berlin patient” [1], the “London patient” [2,3], the “Dusseldorf patient” [3], the “Woman” (or the “New-York patient”) and very recently “The City of Hope” patient reported at the AIDS 2022 conference. Cell transplants are rife with practical challenges including the risk of post-transplant complications (such as pneumonia, sepsis, bleeding, organ failure and chronic graft vs. host disease) and the associated high cost of patient care. Consequently, cell transplants are typically only considered for patients with leukemia or other specific cancers that require extensive radiation and chemotherapy prior to transplantation. Further, the scarcity of compatible donors with the apparent requisite CCR5Δ32 mutation (a host mutation that blocks HIV spread [1,2,3,4]) deems the cell transplantation strategy impractical for the majority of PLWH. Taken together, the challenges associated with this HIV ‘curative’ procedure prevent this cure strategy from feasible implementation and making an impact on the ~38 million PLWH worldwide (WHO, 2020). Hence, a more practical cure strategy is timely. Similarly, gene-editing (CRISPR) has also been considered to permanently purge the provirus leading to a cure. However, gene-editing strategies are limited by the absence of tools or biomarkers to detect the pool of latently infected cells selectively and safely, and the difficulty of using genome editing at a large scale to destroy the provirus [5,6,7,8].

To develop a curative ARV-based treatment for HIV, it is critical to consider the sites of HIV replication persistence whilst adhering to ART. A prominent source of viral rebound is a reservoir of long-lived resting memory CD4^+^ T cells that harbor replication-competent HIV proviruses and appear unextraordinary to host immune surveillance [9,10]. Ongoing HIV replication also persists within various anatomical reservoirs due to specialized immune surveillance mechanisms and the potential to have lower ARV levels relative to the circulating blood/periphery. Cell-to-cell HIV spread and the homeostatic proliferation of infected T cells due to chronic inflammation are further drivers of persistent HIV replication during ART [11]. It follows that a reasonable HIV cure strategy would aim to purge the patient of latently infected cells. This strategy is termed “Shock and Kill”, whereby the intention of the treatment is to reverse proviral quiescence by inducing provirus transcription with pharmaceuticals (the “shock”) and allowing a combination of ART, host immune clearance and HIV-cytolysis to eliminate latently infected cells (the “kill”), which is hypothesized to lead to a complete cure for the patient. However, despite the hundreds of compounds identified as HIV latency-reversing agents (LRAs) to date, none have led to a cure and/or ‘HIV infection remission’ status. Additionally, fundamental clinical and bioinformatics research over the last few decades revealed the complexity and vast heterogeneous nature of HIV provirus reservoirs, casting criticism on the simplicity of the “Shock and Kill” concept in terms of feasibility and reduction to practice. Furthermore, the notion that around 8% of the human genome contains remnants of retroviruses that are no longer expressed (termed ‘human endogenous retroviral sequences/elements’) suggests that the activation of transcription from silenced genes would require higher energy than permanent deactivation [12,13].

The idea of extinguishing HIV replication through the permanent deactivation of provirus transcription has gained momentum over the last decade due to promising in vitro and in vivo results from multiple studies [14,15,16,17,18,19,20,21]. Termed the “block-and-lock” approach, this cure strategy involves ‘blocking’ provirus transcription and “locking” the provirus promoter in a durable silenced state through epigenetic modifications. Such a type of functional cure has been observed in individuals termed post-treatment controllers, who have interrupted their ART and have not observed viral rebound [21]. In these cases, individuals received ART very soon after infection, which is atypical, given that many individuals are not immediately aware of their HIV status following exposure. Post-treatment controllers are rare, about 5 to 15 percent of people living with HIV [21], and it is not fully understood how these individuals maintain viral suppression in the absence of ART. However, some controllers have proviruses in deep transcriptional dormancy by encapsulating their proviruses in heterochromatin regions [14]. The block-and-lock approach seeks to mimic this viral suppression, in the first instance using novel small molecules to epigenetically silence HIV, followed by the removal of all drugs, allowing for an ART-free life.

To expand on the “block-and-lock” type of approach by targeting viral or either host transcriptional factors and chromatin regulators, it is important to have a deep understanding of the mechanisms regulating HIV-1 latency and reactivation in memory CD4^+^ T cells. Here, we describe and review recent advances in the development of HIV-1 transcription regulation and transcription inhibitors that could benefit ARV-based HIV cure efforts.

## 2. Regulation of HIV-1 Transcription

### 2.1. HIV Transcription

**Figure 1:**
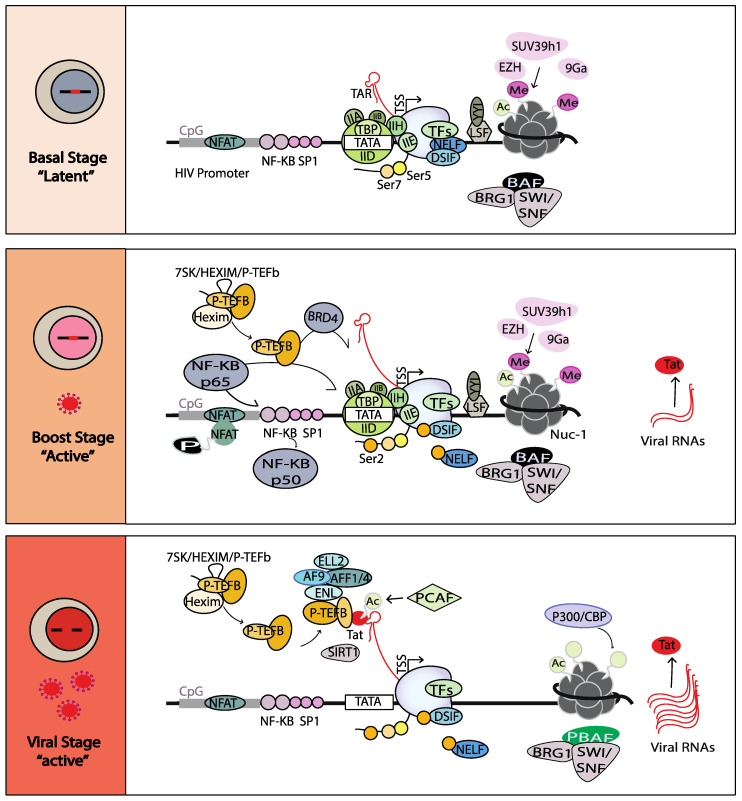
Basal, Boost and Viral stages of HIV transcription.

HIV-1 transcription is regulated by three different phases based on a temporal interplay between host and viral factors in the HIV promoter (Figure 1) [22,23,24]. It is initiated with the recruitment of host transcription factors, such as Nuclear Factor-κB (NF-κB) and SP1, and other general transcription factors, such as transcription factor IID, IIA, IIB, IIE, IIF and IIH (TFIIH), to their cognate sites on the HIV promoter, forming the pre-initiation complex (PIC). These factors allow the recruitment of the hypophosphorylated RNA polymerase II (RNAPII) at the HIV promoter [25]. The ATP-dependent DNA helicase XPB, a subunit of TFIIH, then facilitates negative DNA supercoiling that is threaded through the RNAPII active site. The cyclin-dependent kinase 7 (CDK7) subunit of TFIIH next phosphorylates Ser 7 and Ser 5 of the RNAPII C- terminal domain (CTD), activating the RNAPII [26]. During this phase, transcriptional elongation is not efficient and rapidly aborts due to the scarcity of positive modulators sequestrated in an inactive form (such as NF-κB and the positive transcription elongation factor (P-TEFb)) [27,28], the presence of transcriptional repressors (such as the negative elongation factor (NELF)), DRB sensitivity inducing factor (DSIF), Yin Yang 1 (YY1) and the C-promoter binding factor (CBF)) [29], the positioning of nucleosome-1 (Nuc-1) downstream of the transcription start site (TSS) [19] and the presence of negative chromatin remodelers [30,31]. Only short viral transcripts of 60 nucleotides (nascent TAR RNA) downstream of the TSS are synthetized and accumulated [32]. This initial step is designated as the “basal” state of provirus transcription [33].

As a result of immune activation, transcription factors, such as NF-κB and Bromodomain-containing protein 4 (BRD4), translocate into the nucleus [34,35] and facilitate inefficient recruitment of P-TEFb (a complex formed by Cyclin T1 and CDK9). P-TEFb phosphorylates the transcriptional repressors NELF and DSIF, along with the RNAPII CTD at the Ser 2 residue. NELF is then released from the HIV promoter and DSIF is converted into a positive transcription factor. It has also been reported that BRD4 might act as a kinase that could further phosphorylate the Ser 2 residue of RNAPII [36]. Ultimately, full-length HIV mRNAs are transcribed, spliced and translated to produce the immediate/early HIV proteins, including the “Trans-Activator of Transcription” protein known as “Tat”. This second level of activation is designated as the “boost” state of provirus transcription [33].

When the Tat protein level reaches a certain threshold, the Tat protein efficiently induces exponential transcription elongation of the provirus. Tat protein binds to the TAR RNA through its arginine-rich domain. The histone acetyltransferase (HAT) p300/CBP-associated factor (PCAF) acetylates Tat on K 28 residue to mediate P-TEFb recruitment to the Tat-TAR complex, previously released from an inactive complex with 7SK snRNP-HEXIM [37]. Tat also recruits the super elongation complex (SEC) to the HIV-1 promoter, including a scaffold protein (AFF1/4), co-factors (ENL and AF9) and the positive elongation factor ELL2 [38,39]. P-TEFb and ELL2 synergistically activate the RNAPII. It was shown that the SEC and Tat protein stabilize ELL2, which helps to robustly activate the RNAPII [40]. HATs acetylate or crotonylate histones, leading to an open chromatin environment and the recruitment of the SWI/SNF chromatin-remodeling complex, polybromo-associated BAF (PBAF). PBAF repositions Nuc-1 further downstream of the TSS to allow transcription elongation [41,42]. Conversely, the BRG1- or HBRM-associated factors (BAF) complex, which generated repressive chromatin conformation, is released from Nuc-1 [43,44]. Finally, the Tat protein is deacetylated by the sirtuin 1 (SIRT1), a member of the class III histone deacetylase (HDAC) family, releasing it from the RNAPII. The Tat protein is then recycled and recruited to TAR for a new cycle of HIV transcription [45]. This phase is designated as the “viral phase” of provirus transcription [33].

### 2.2. Mechanisms of HIV-1 Latency and Potential Therapeutic Targets

Despite intensive research, the molecular dynamics of HIV-1 latency regulation are still incompletely understood. Studies in in vitro and in vivo showed multiple mechanisms of HIV-1 latency, including epigenetic modulation, transcriptional interference, the sequestration of transcription factors and limited Tat levels [22,24,27,30,43,46,47,48,49]. These studies inspire the development of tools and targets to enhance current novel therapeutic strategies [50,51].

#### 2.2.1. Epigenetic Modifications and Modulation

Studies have shown that there are three main types of epigenetic processes that impact HIV transcription: (a) the positioning and remodeling of nucleosomes along the genome; (b) histones modifications and (c) DNA modifications. The intricate and cooperative balance between these epigenetic processes significantly impacts HIV latency. Indeed, the HIV provirus is organized into chromatin. The structural repeating unit of chromatin is the nucleosome, whereby DNA is wrapped around an octamer that comprises pairs of four-core histone proteins (H2A, H2B, H3 and H4) [52]. The latent provirus promoter contains three nucleosomes (Nuc-0, Nuc-1 and Nuc-2), which flank two DHS regions [53]. The level of the condensation of the chromatin defines the accessibility of factors to the DNA sequence, and thus, the extent of HIV transcription. The accumulation of condensed chromatin over time is likely the main driver of provirus transcription silencing [14].

##### Positioning and Remodeling of the Nucleosomes

The ATP-dependent chromatin remodelers, grouped into five classes [54], diminish DNA/histone interactions critical to repositioning or restructuring nucleosomes. In particular, the BAF complex can be selectively recruited to the HIV promoter by the short isoform of BRD4 [55]. The BAF complex then allows the precise positioning of Nuc-1 immediately downstream of the TSS, reinforcing HIV transcriptional repression [56,57]. Upon viral reactivation, BAF dissociates from the HIV promoter and is replaced by the PBAF complex via Tat-dependent [58,59] or -independent mechanisms [60]. This results in the re-positioning of the nucleosomes to energetically more favorable positions for unlocking provirus transcription and efficient transcript elongation [44,59]. The important role of the BAF complex in HIV repression leads to studies investigating small molecules targeting BAF complexes for HIV latency reversal [61]. The NURD/Mi-2/CHD complex has also been linked to HIV latency. Both CHD1 and CHD2 were shown to be essential to the regulation of HIV transcription, yet the underpinning molecular mechanisms are unknown [62,63]. Additionally, specific histone chaperones complexes might be involved in finely tuning nucleosome occupancy and HIV gene expression, such as the HIRA (Histone Cell Cycle Regulator) and the FACT (Facilitates Chromatin Transcription) complexes involved in HIV latency regulation [62].

##### Histone Modifications

Histone-reversible covalent modifications by specific enzymes negatively regulate HIV-1 transcriptional activity by altering histone affinity to DNA. Additionally, histone changes were shown to regulate transcription by serving as a scaffold for the binding of effector proteins [64]. These crucial functions of the histone covalent modifications led to the notion of a “histone code” in the early 2000s [65]. The most well-characterized histone modifications at Nuc-1 are HDACs, histone methyltransferases (HMTs) and histone acetyltransferases (HATs). They regulate HIV transcriptional activity and change the ability of RNAPII to initiate transcription. Histone deacetylation is mediated by HDAC enzymes that erase the acetylation of lysine ε-amino groups [66]. Multiple repressive host transcription factors were reported to allow the recruitment of class-I HDACs during HIV latency [67,68,69,70,71], such as the YY1 factor and the transcription factor CBF-1 [71]. Using a mechanism independent of histone deacetylation, the class-III HDAC SIRT1 was shown to control the recycling and the transactivation feedback of Tat [46]. In contrast to the well-characterized positive effects of acetylation, a recent interesting report showed that lysine acetyltransferase 5 (KAT5) promotes HIV latency through the acetylation of histone 4 on the HIV promoter, allowing the recruitment of BRD4 and the inhibition of HIV transcriptional elongation [72]. This latter study demonstrated that the hypoacetylation of histone 3 and the hyperacetylation of histone 4 have an opposite compatible role in the heterochromatinization of the HIV promoter during latency [72]. Understanding the mechanisms modulating the histone acetylation patterns or “histone code” is of immediate urgency in the context of ‘shock and kill’ cure strategies, given their potential to instruct the rational development of specific, potent, and safe HIV latency reversal agents.

The methylation of histone tails involves the addition of 1–3 methyl groups, either on lysine residues by histone lysine methyltransferases (HKMTs) or on arginine residues by protein arginine methyltransferases (PRMTs) [73]. The HMT EZH2, which is part of the Polycomb Repressive Complex 2 (PRC2), was shown to induce H3K27me3, resulting in latency of the viral promoter in cell lines and primary cell models [74]. In addition, both EHMT1 (Euchromatin histone methyltransferase)/GLP and EHMT2/G9a participate in HIV latency by depositing H3K9me2 on the HIV promoter in latently infected T cell lines [75,76,77]. A recent report has identified the transcription factor CBF-1 as responsible for the combined recruitment of these HMTs and HDACs, further supporting the notion that histone methylation and deacetylation are coordinated in HIV silencing [78]. Regarding other histone lysine residues, SMYD2 (SET and MYND Domain Containing 2) has been involved in HIV latency by mediating the mono-methylation of H4K20me, which could then potentially lead to the recruitment of the PRC1 and further chromatin compaction [79]. Similarly to the histones, a recent study has shown that histone demethylases are also involved in HIV latency. Indeed, the histone demethylase MYC-induced nuclear antigen (MINA53), identified in a CRISPR/Cas9 screen, facilitates HIV promoter heterochromatinization through the demethylation of H3K36me3 [80]. Thus far, only one study reported the involvement of histone arginine methylation in HIV promoter silencing using CARM1/PRMT4 catalyzing H3R26 methylation [80]. Finally, a recent study has shown that histones can be hypocrotonylated in latently infected CD4^+^ T cells [81]. Histone crotonylation consists of the addition of a crotonyl group onto lysine ε-amino groups using crotonyl-CoA as a cofactor [64]. Accordingly, the hypocrotonylation of the HIV promoter in latent cells was correlated with a lower expression of Acyl-coenzyme A synthetase short-chain family member 2 (ACSS2), an enzyme that participates in the synthesis of crotonyl-CoA [81]. This study was further supported by the aberrant fatty acid metabolism linked in HIV^+^ individuals with low ACSS2 expression, potentially favoring the establishment of HIV latency [81]. Collectively, a comprehensive picture of the dynamic modulation of the histone code of HIV latency is currently lacking. Future studies on the crosstalk between histone marks would be beneficial to the block-and-lock functional cure strategies.

##### DNA Methylation

DNA methylation typically functions to repress gene transcription, and numerous studies provide empirical data supporting the link between HIV latency and the DNA hypermethylation of CPG islands flanking the HIV TSS [82,83,84,85,86]. DNA methylation is catalyzed by specific and multiple DNA methyl transferases (DNMT) [47], whereby the admdition of a methyl group on the fifth carbon of the cytosine pyrimidine ring with the CpG islands results in a molecular environment that does not favor transcription [82,87,88]. The duration of HIV latency is contingent on the coordination of multiple DNMTs to maintain specific DNA methylation patterns of the provirus [89,90]. For example, changes in the hypermethylation state of the provirus promoter were positively associated with the infection duration (and ART efficacy) [90,91]. However, the underpinning mechanisms contributing to HIV promoter methylation over time remain to be elucidated in the context of drug discovery and development.

Collectively, the mechanism of the dynamic and collaborative changes in the HIV nucleosome array during latency establishment, maintenance and reversal remains to be determined and will likely bridge several mechanisms. Additionally, both cellular and HIV-transcribed non-coding RNAs (ncRNA) might be exploited to silence HIV transcription [92,93]. These recent observations add yet another layer of complexity when considering chromatin architecture containing provirus to further promote epigenetic repression.

#### 2.2.2. Transcription Interference

It was demonstrated that silenced HIV preferentially integrates into the introns of actively transcribed host genes through global transcriptional profiling studies using human T cell lines [94,95]. These findings were confirmed in primary cells and resting CD4^+^ T cells from an individual living with HIV [96,97]. This could be explained by the suppression of one transcription unit by another neighboring cis-element called ‘transcription interference’ [98]. Indeed, active neighboring promoters can directly repress or interfere with the HIV promoter transcription. The type of interference depends on the orientation of HIV relative to the host gene. When a host gene polymerase positioned upstream of the provirus reads through the HIV promoter, transcriptional interference can occur by promoter occlusion, causing the dislodgement of necessary transcription initiation or elongation complexes. Alternatively, convergent transcription aborts viral expression when the proviral and the host gene RNAPII complexes are in opposite orientations and collide [50,99,100]. Studies assessing transcription interference in multiple HIV latent cell models, including ACH2 and J-Lat cells, and in cells from ART-suppressed individuals, revealed greater transcriptional interference in ACH2 and J-Lat cells from the transcriptionally active upstream host gene [100,101], resulting in human/HIV hybrid transcript expression that mostly terminated within the 5′ LTR. These studies highlight the limitations of using cell lines to recapitulate HIV latency. However, Winecoff et al., recently reported that transcriptional interference moderately interfered with HIV transcription in different cell models without affecting the response to latency reversal agents, suggesting a minor role of transcriptional interference in the maintenance of latency [102].

#### 2.2.3. Sequestered Cellular Transcription Factors

Latency can be promoted by multiple mechanisms, including the lack of host transcription factors, elongation factors [103,104], or the enrichment of transcriptional repressors to the HIV promoter [105,106]. Latent memory CD4^+^ T cells with suboptimal HIV transcription were reported to have transcription activators sequestered in an inactive state, contributing to the silencing of their HIV transcription. For instance, under resting conditions, NF-κB (p50/p65 heterodimers) is sequestered in the cytosol, tightly bound to I*κ*B, along with p50/p50 homodimers occupying the NF-*κ*B sites and recruiting HDACs, creating a repressed cellular environment. Upon the cytokine activation and phosphorylation of the p65 subunit, p65 is released from I*κ*B and translocates to the nucleus, where it binds to the NF-*κ*B sites in the HIV-1 promoter, leading to viral transcription [107,108,109]. Therefore, targeting different steps of the NF-κB pathway was explored for HIV transcription inhibition (see Section 3.2.2). Similarly, the transcriptional activator NFAT has been demonstrated to be sequestered in the cytosol. Upon cellular activation by calcium signaling, NFAT is dephosphorylated by calcineurin phosphatase and translocated into the nucleus where it binds to the NF-κB sites in the HIV promoter, leading to HIV activation [110,111].

As mentioned above, the host transcription factor P-TEFb plays a key role in the regulation of HIV transcription. Thus, the scarcity of its active form would affect HIV transcriptional elongation [51,112] (see Section 3.2.1). P-TEFb incorporates into an inactive inhibitory 7SK snRNP complex containing HEXIM and 7SK snRNA, by the direct binding of Cyclin T1 to HEXIM, preventing efficient HIV transcription elongation [19]. When the HIV Tat interacts with Cyclin T1, it results in the dissociation of P-TEFb from the 7SK snRNP complex [49,113,114,115].

Finally, numerous other host cellular factors regulating HIV-1 transcription being sequestered have been reported, including AP-1 [116], SP1 [117], CDK2 [118,119], P300/CBP [119], C/EBP [120], TCF/LEF-1 [121], IFI16 [122], Ssu72 [123], ELL2 [40], AFF4 [39],TFIIH [124,125], KAT5 [72], mTOR complex [126], ITK [127], ABIN1 [128], DYRK1A [129], LSF and YY-1 [105]; c-Myc [130]; CTIP-2 [131]; CBF-1 [71]; FTSJ3, TMEM178A, NICN1 and the integrator Complex [132]. A deep understanding of their mechanism of action would allow to design optimal combinatorial strategies to specifically either reverse viral latency in infected resting CD4^+^ T cells and render cells visible to immune surveillance or to block any activation by further amplifying their sequestration.

#### 2.2.4. Role of HIV Tat

Once integrated into the host chromosome, the HIV-1 becomes subject to regulation by host and viral transcription factors [133,134,135,136]. The HIV Tat protein plays a crucial role in the transition between HIV latency to activation [48]. Once the amount of Tat protein accumulates to a critical level, sequence-specific interactions of Tat protein with host factors and the TAR RNA result in the recruitment of a myriad of host factors to assure high-level, productive transcription [137,138,139]. This cycle of amplification is called the Tat positive feed-forward loop.

**Figure 2:**
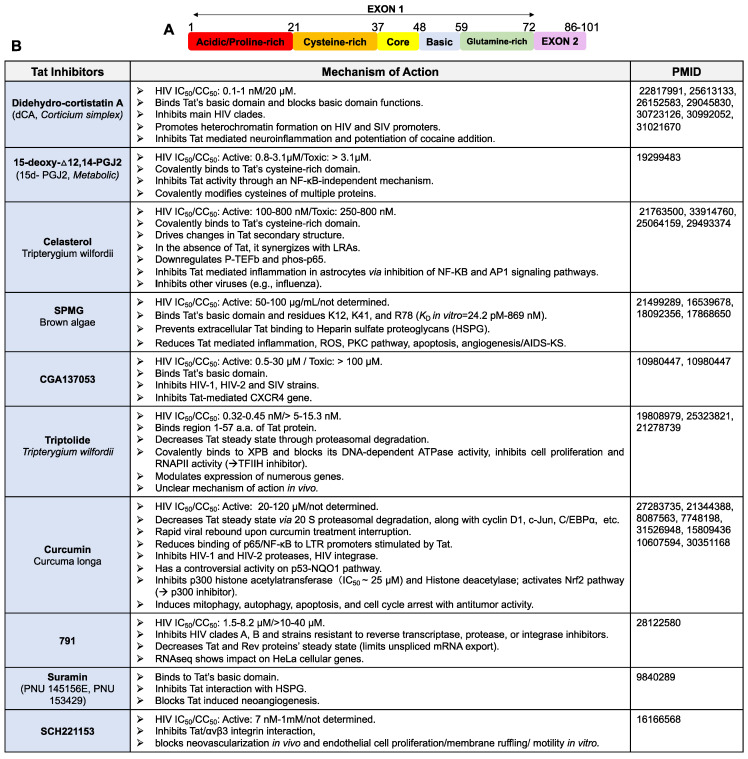
Tat inhibitors. (**A**) Schematic of Tat protein’s domains. (**B**) Inhibitors of HIV Tat protein activities.

The HIV Tat protein is a small flexible protein of 86 to 103 amino acid residues. It is encoded by two exons and divided into six functional regions (Figure 2A). The N- terminal domain (1–48 aa), which includes an acidic region (residues 1–21), a cysteine-rich region (residues 22–37) and a hydrophobic core region (residues 38–48), is critical for HIV transcriptional activation [140]. The cysteine-rich region with seven conserved cysteines (except for HIV subtype C) is required for Tat structure stabilization, metal binding and interaction with CyclinT1. The arginine-rich or basic region (49–57 aa) is the well-conserved sequence ^49^RKKRRQRRR^57^ and it is critical for Tat cellular and nuclear membrane translocation, interaction with multiples partners including the HIV TAR RNA [140,141,142] and Tat-mediated inflammation [143]. Next, the region V (residues 60–72) is the glutamine-rich region and it has the highest rate of sequence variation. It has been linked to Tat-induced apoptosis [140,144,145]. Finally, Region VI constitutes the C-terminus of Tat and is encoded by the second exon [140]. It is mostly important for the HIV replication in both T cells and macrophages, along with the uptake of Tat via its RGD motif for the HIV clade B and D. Blocking Tat expression or preventing Tat interactions with its molecular partners was shown to modulate the level of viral activation in different models of HIV latency [146].

## 3. Promising HIV Transcription Inhibitors

A potent host or viral transcriptional inhibitor small molecule candidate should display favorable pharmacokinetics characteristics such as a good drug-like structure, good solubility, long-lasting activity and a reasonable large-scale cost of production [147]. In addition, these should also display biological characteristics such as no cytotoxic/off-target activity, a high selectivity index, a high barrier for resistance development, inhibition of viral production from integrated viral genomes and consequently promote the epigenetic silencing of HIV transcription over time, limiting reactivation from latency in the absence of ART.

### 3.1. Targeting HIV Tat or TAR

#### 3.1.1. Tat Inhibitors

The HIV Tat protein is a very attractive target for therapeutic intervention since Tat is expressed early during virus replication and has no host cellular homologs; thus, Tat inhibitors can efficiently and specifically reduce viral production without affecting cellular transcriptomics. Contrary to current antiretrovirals that block de novo infection, Tat inhibitors can block transcription from the integrated provirus, which over time may result in the long-lasting epigenetic silencing of the HIV promoter, which becomes resistant to reactivation [119]. Tat inhibitors may also reduce Tat-mediated HIV Associated Neurocognitive Disorders (HAND) or other Tat-mediated pathologies [148]. Importantly, we suspect that the barrier to the evolution of viral resistance may be very high, given the key role played by Tat in HIV expression and that mutations in Tat or TAR are often associated with a drastic loss of transcriptional fitness. An ideal Tat inhibitor should block the Tat-mediated activation of the viral promoter without affecting host cellular homeostasis and target Tat from the variety of HIV clades. It is important to note that during acute HIV infection, anti-Tat molecules would not be able to completely inhibit replication, given that the initial round of transcription is triggered by the cellular transcription machinery. However, these molecules would have enormous potential if added to the current ARV ammunition and especially, as we mentioned above, in functional cure approaches.

Two types of Tat inhibitors have been described (Figure 2B): (1) Tat binders that may or may not modify its structure and interfere with its molecular partner interactions (such as didehydro-cortistatin A, dCA [18,149]), or (2) Tat degraders (such as Triptolide) [119,146,150,151,152,153]. In a general manner, therapeutics that degrade Tat might be more efficient than specific Tat inhibitors. Tat degraders would limit all or most possibilities of Tat/host protein interactions, presumably resulting in a more definitive interruption of the positive feed-forward transcription loop relative to interrupting one or two of many Tat interactions with molecular partners.

Tat may be degraded through different pathways, including the lysosomal pathway [154], the ubiquitin-independent 20S proteasomal pathway [155,156], the lysine 48-linked ubiquitin chain, which is then recognized by the 26S proteasome pathway [157] or by autophagy [154]. Multiple host proteins were shown to impact the Tat protein’s half-life. Indeed, the tumor-suppressor proteins p14^ARF^ can inhibit Tat transactivation by promoting Tat degradation through a ubiquitin-independent pathway [158]. The lncRNA NRON or the host cell E3 ubiquitin ligase protein CHIP directly link Tat to the 26S proteasome components [159,160]. As for the CycT1-U7, a CyclinT1 mutant protein, it was shown to induce a dominant negative effect on HIV transcription by promoting Tat degradation [161]. Finally, USP21 inhibits HIV production by specifically downregulating Tat expression by deubiquitinating Tat, causing Tat instability and reducing CyclinT1 mRNA levels [162]. Viral proteins were also shown to affect Tat expression, such as the nucleocapsid and Rev protein [155,163]. In contrast, Tat was reported to be stabilized by USP7 and PRMT6 [164,165].

##### Tat Binder: Didehydro-Cortistatin A (dCA)

dCA is an analog of a natural steroidal alkaloid from marine sponge and a potent and selective Tat inhibitor. dCA potently inhibits HIV-1 production in acutely and chronically infected cells at subnanomolar concentrations, as well as in primary CD4^+^ T cells, without cell-associated toxicity [18]. By binding specifically to the TAR-binding domain of Tat, dCA disrupts the Tat-TAR interaction, resulting in the inhibition of Tat-mediated transactivation and transcriptional amplification [18,149]. It was also demonstrated that dCA limited Tat-induced inflammation in cell models, as well as cocaine drug addiction potentiation in mice [18,148,149]. It was demonstrated that dCA led to a tighter nucleosome/DNA association to the HIV promoter in several cell models of HIV latency, without impacting classic nucleosome positioning in the HIV promoter. The SWI/SNF chromatin remodeling complex PBAF and the RNAPII recruitment on the HIV genome were restricted, driving viral gene expression into a durable state of latency, refractory to HIV reactivation by current LRAs [16,18,166]. The specificity of dCA to Tat was supported by the lack of the activity of dCA in latent cell models with a deficient Tat-TAR axis [16]. In human primary CD4^+^ T cells from aviremic-infected individuals, the long-term treatment of dCA led to long-lasting HIV silencing. It prevented viral rebound after treatment interruption in the presence of strong cellular activators. In the bone marrow-liver-thymus (BLT) mouse model of HIV latency and persistence, a combination of dCA to ART for 14 days resulted in a loss of viral RNA in multiple HIV reservoirs, including the brain where dCA was shown to easily cross the blood-brain-barrier. Furthermore, combining dCA with ART for a period of 4 weeks significantly accelerated HIV suppression and slowed down viral rebound upon treatment interruption [15]. Finally, a resistance study recently reported an absence of mutations on TAR or Tat, emphasizing the high genetic barrier to dCA resistance [167]. While dCA makes a compelling case for advancement into clinical trials [168,169,170,171], the cost of large-scale production remains critical.

##### Tat Degrader: Triptolide

Triptolide is a natural product isolated from Tripterygium wilfordii Hook F, a traditional Chinese herb, known for its anti-inflammatory, immunosuppressive and anti-tumor properties [172]. The anti-HIV activity of Triptolide was first reported by Wan et al. [152], showing a remarkable picomolar/*nanomolar* inhibition of HIV replication in cell lines and peripheral blood mononuclear cells. While Triptolide did not alter the stability of Tat mRNA, it reduced Tat steady-state protein levels via the proteasomal pathway. Mutagenesis studies revealed that Tat residues 1 to 59 are required for the Triptolide-mediated degradation. Triptolide pharmacological activity and chemical synthesis pathways along with toxicological and clinical studies have been discussed in detail elsewhere [173]. Unfortunately, Triptolide presents multiple off-target activities, displays poor solubility and shows high toxicity, strongly narrowing its therapeutic capabilities. Indeed, Triptolide was shown to interfere with the TNF-α-induced NF-κB activation [174] and inhibit RNAP I, II and III activities [175,176,177]. Besides, Triptolide inhibits nucleotide excision repair by covalently binding to the ATP-dependent DNA helicase XPB, a subunit of TFIIH, and preventing its DNA-dependent ATPase activity [178]. Recently, Triptolide was reported to block RNAP III transcription in colorectal cancer cells by directly disrupting the formation of the transcription factor TFIIIB [177]. Despite the concerns mentioned above, the effect of Triptolide on the HIV reservoir is currently tested in phase III clinical trials (NCT02219672 and NCT03403569).

#### 3.1.2. TAR Inhibitors

The highly conserved HIV TAR RNA, present at the 5′ of all HIV messengers, plays a crucial role in the HIV life cycle, including its key interaction with Tat [179,180]. In a general manner, TAR inhibitors disturb the Tat–TAR interaction through competition or allosterically by directly binding to the TAR RNA three-base bulge region or the three-base bulge together with the lower and upper-stem/Loop region [153]. For instance, WM5, a 6-aminoquinolone, binds to the bulge of TAR and suppresses Tat-mediated LTR activity and viral replication [181]. With the advances in high throughput technologies and RNA biology and function, new screening methods allowed the discovery of more potential hits targeting TAR, including small molecules, peptides and evolved proteins [182,183,184]. However, their low solubility and high toxicity have so far hampered their clinical development.

### 3.2. Targeting Host Factors

Targeting host transcriptional factors may on the one hand provide an increased threshold to viral resistance evolution; however, these may negatively affect cell survival and homeostasis, thus the therapeutic window must be carefully studied. In this section, we will describe a few host transcriptional inhibitors that play important roles in HIV transcriptional regulation and have the potential to be druggable. A comprehensive list of inhibitors may be found in Table 1 and Table 2.

#### 3.2.1. P-TEFb Inhibitors

P-TEFb plays essential roles in transcriptional elongation [27,114,185]. The crystal structure of the CDK9/Cyclin T1/Tat complex [186,187] offers the possibility to design inhibitors targeting specifically the interface of this viral–host complex [147,188]. Approaches to manipulate P-TEFb for transcriptional inhibitors’ development are ongoing and include the inhibition of CDK9 kinase activity, neutralizing Cyclin T1 or its interaction with Tat, shifting P-TEFb equilibrium, changing P-TEFb protein levels and modulating the interaction between P-TEFb and its recruitment factors [151,161,188,189,190,191,192,193,194,195,196,197,198]. Thus far, P-TEFb inhibitors lack specificity to HIV transcription, and often result in undesired toxicity [188].

Since P-TEFb functions depend on the kinase activity of CDK9, targeting CDK9 has been extensively studied. The most characterized first-generation CDK9 inhibitor is Flavopiridol, which competes with ATP for CDK9′s catalytic site at low nanomolar concentrations [190], inhibiting HIV Tat-transactivation. A series of Flavopiridol analogues has been developed to improve selectivity and reduce toxicity [189,191,192]. Structural biology approaches allowed the development of second and third generations of CDK9 inhibitors, such as F07#13 [198], CR8#13 [151], CYC202 [193] and IM [196]. These inhibitors were optimized to specifically target HIV-1 transcription/replication with low or no toxicity. Increasing evidence indicates that many new CDK9 inhibitors including Dinaciclib, BAY1143572, P276-00 and TG02 may also be used in cancer treatment [188]. It is also worth mentioning that one study showed that the nature of the strategy to inhibit CDK9 profoundly affects the patterns of gene expression resulting from CDK9 inhibition, suggesting multiple variables affect the outcome, including the kinetics of inhibition, potency, off-target effects and selectivity. This is especially important when considering CDK9 inhibition for therapeutic purposes.

As for Cyclin T1, Cyclin T1 intrabodies [194], Cyclin T1-dominant negative mutants [161], microRNA-198 [197] and the C3 compound were developed [195]. C3 presents low cytotoxicity and limits Tat binding to Cyclin T1, resulting in suppression during acute HIV replication and reactivation from latency. This compound was shown to suppress Tat-mediated HIV LTR-driven gene expression and RNAPII phosphorylation. Furthermore, molecular docking studies revealed the interaction of C3 with the Tat-binding amino acids of Cyclin T1 [195].

**Table 1:** P-TEFB inhibitors (part 1), P-TEFB inhibitors (part 2).

P-TEFB INHIBITORS		MECHANISM OF ACTION	PMID/References
**CDK9**	**Flavopiridol** (Alvocidib)	➢ CDK inhibitor potent against CDK1, 2, 4, 6, 7, and 9. ➢ Change conformation of CDK9 to disable binding to ATP. ➢ Broad-range cellular effects including transcriptional inhibition by blocking RNAPII phosphorylation, promoting apoptosis, anti-angiogenesis and cellular arrest. ➢ Promotes loss of P-TEFB that correlates with a reduction in HIV replication. ➢ Its anti-HIV activity varies from IC_50_: 6–61 nM and toxicity CC_50_: 99-225 nM.	10665481, 11013232, 19509270, 16204078, 10559866, 15150125, 17625008, 23092279, 29471852
	**Seliciclib** (Roscovitine, CYC202)	➢ ATP competitor inhibits the human CDK2/cyclin E, CDK1/cyclin B, CDK7/cyclin H and CDK9/cyclin T1, and weakly CDK4, CDK6 and CDK8. ➢ Through inhibition of CDK7 and CDK9, blocks RNAPII phosphorylation leading to suppression of viral reactivation in HIV latent cell lines. ➢ Reduces acute HIV replication from T-tropic, monotropic and dual tropic viral strains, along with resistant strains. ➢ Anti-HIV activity varies from IC_50_: 0.36-35 μM. ➢ Reduces CDK2-cyclin E and P-TEFB present at the HIV genome, ➢ Blocks degradation of p53 through the inhibition of MDM2 expression, induces caspase-dependent apoptosis, downregulates the antiapoptotic proteins Mcl-1 and XIAP, and IL-6, upregulates Bak expression and Bax cleavage, impacts cell cycle. ➢ Acts on multiple diseases (e.g., cancer, leukemia, HSVs).	17179992, 25747275, 15531588, 17625008, 29471852
	**DRB**	➢ Inhibits the CTD of kinases including CDK2,7, 8 and 9. ➢ Binds the ATP binding site of CDK9. ➢ Displays ≥ 25-fold selectivity for CDK9 over both CDK7 and CDK2 in vitro. ➢ Induces a loss of P-TEFB and inhibits RNAPII phosphorylation, blocking HIV transcriptional elongation and Tat-mediated transcription with IC50 of 2.6-5 μM. ➢ Inhibits influenza virus multiplication.	23092279, 17625008
	**PHA-767491** (CAY10572)	➢ Is an ATP-competitive dual inhibitor CDC7/CDK9 with IC_50_ of 10 nM and 34 nM in in vitro assays, respectively. Inhibits CDK1/2 and GSK3-β with ~20-fold less selectivity, MK2 and CDK5 with 50-fold less selectivity and PLK1 and CHK2 with 100-fold selectivity. ➢ Prevents initiation of DNA replication, cell proliferation and induces apoptosis in a p53-independent manner. ➢ Impedes TCR signaling, suppresses T cell activation/responses, proliferation, and effector functions, which could be detrimental for the immune response. ➢ Showed severe adverse effects in several early-stage trials.	26766294,18469809, 20197552, 21768328, 31402912, 31402912
	**BAY1143572** (Atuveciclib)	➢ P-TEFb/CDK9 inhibitor with IC_50_=13 nM for CDK9/CCNT1 and the ratio of IC_50_ values for CDK2/CDK9 is about 100. It also inhibits GSK3 kinase with IC_50_ values of 45 nM and 87 nM for GSK3α and GSK3β respectively. ➢ Inhibits RNAP II (Ser2) phosphorylation and downregulates MYC protein expression. ➢ Antiproliferative activity.	28961375, 29471852
	**LY2857785**	➢ Competitive ATP kinase inhibitor against CDK9 (IC_50_: 0.011 μM) but also inhibits CDK8 (IC_50_: 0.016 μM), CDK7 (IC_50_: 0.246 μM), along with CDK4, CDK6, and CDK2, CDK1 (IC_50_: 0.241 μM) enzymatic activities. ➢ Inhibits cellular RNAPII CTD Ser2 and 5 phosphorylation at IC_50_s 0.089 and 0.042 μM. It does not induce G1-S cell-cycle arrest, only a moderate G2–M DNA content increase. ➢ Blocks hematologic and solid tumor cell proliferation, reduces levels of MCL-1, leading to apoptosis in vitro.	29471852, 24688048
	**Dinaciclib** (SCH727965)	➢ Inhibitor of CDK1, 2, 5 and 9 with IC_50_ of 3 nM, 1 nM, 1 nM, and 4 nM, respectively. ➢ Blocks thymidine DNA incorporation (IC_50_: 4 nM) and suppressed retinoblastoma phosphorylation, which correlated with induction of apoptosis. ➢ Induces cell-cycle arrest in more than 100 tumor cell lines. ➢ Broad antiproliferative activity, downregulates expression of MCL-1 and induces apoptosis in in vitro and in vivo models of leukemia, significantly prolonged survival in vivo. ➢ Evaluated in clinical trials, alone or in combination, in various hematologic indications, with varied efficacy and side effects.	29471852
	**Voruciclib** (P1446A-05)	➢ Is an inhibitor of CDK9/CCNT2, CDK9/CCNT1, CDK6/CCNT D1, CDK4/CCNT D1, CDK1/CCNT B, and CDK1/CCNT with IC_50_ of 0.626 nM, 1.68 nM, 2.92 nM, 3.96 nM, 5.4 nM, 9.1 nM, respectively. ➢ Its inhibition of CDK9 leads to decreased expression of RNAPII transcriptional targets such as MYC and MCL1. It results in reduced phosphorylation of MYC and total MYC protein, leading to the inhibition of cellular growth in multiple KRAS mutant cancer in in vivo and in vitro. Voruciclib represses expression of MCL-1 in multiple models of diffuse large B-cell lymphoma. ➢ Though its inhibition of CDK4 and CDK6, it induces cell cycle arrest, suppresses DNA replication and decreases tumor cell proliferation. ➢ Is being evaluated in clinical trial on patients with B-cell malignancies and acute myeloid leukemia.	29269870 *Abstract. Cancer Res* (2021) 81 (13_Supplement): 1962.
	**SNS-032** (BMS-387032)	➢ Was initially described as a selective inhibitor of CDK2 with IC_50_ of 38 nM in in vitro and is 10- and 20-fold selective over CDK1/CDK4. It was then found to inhibit CDK7/9 activities with IC_50_ of 62 nM/4 nM, with small effect on CDK6. ➢ Blocks cell cycle *via* inhibition of CDK2 and 7, and transcription *via* inhibition of CDK7 and 9, resulting in apoptosis. ➢ Induces a dephosphorylation of Ser 2 and 5 of RNAPII and inhibits the expression of CDK2 and CDK9 and dephosphorylates CDK7. ➢ Showed a high inhibition of T cell activation marker expression, exceeding that of PHA-767491. ➢ Is being evaluated in clinical trial alone or in combination on patients with chronic lymphocytic leukemia and multiple myeloma, both B cell malignancies. Adverse effects were observed.	21212792, 19169685, 29471852, 31402912
	**P276-00** (Riviciclib)	➢ Is a CDK1, 4 and 9 inhibitor with IC_50_ of 79 nM, 63 nM and 20 nM, respectively. ➢ Shows antiproliferative effects against various human cancer cell lines, down-regulates cyclin D1 and CDK4 in an ATP-competitive manner and decreases CDK4-specific retinoblastoma protein phosphorylation, induces apoptosis by activating cellular caspase-3 activity and DNA ladder formation. ➢ Induces apoptosis that correlates with transcription inhibition and a significant decline in Mcl-1 protein levels with the appearance of cleaved PARP in myeloma cells. In vivo studies confirmed its antitumor activity.	17363486, 29471852
	**TG02** (Zotiraciclib)	➢ Is an inhibitor of several CDKs (CDK9: 3 nM, CDK5: 4 nM, CDK2: 5 nM, CDK3: 8 nM, CDK1: 9 nM, CDK7: 37 nM) together with JAK2 and FLT3. ➢ Displays antiproliferative effects against tumor cell lines, induces cell-cycle arrest and apoptosis in leukemia cells, and prolongs survival in murine acute myeloid leukemia models. ➢ Inhibits transcription by inducing RNAP II Ser2 dephosphorylation and downregulates MCL-1 and XIAP, resulting into BAX activation and apoptosis. ➢ Is being evaluating in advanced hematologic malignancies and brain tumor.	29471852, 21860433
	**AZD4573**	➢ Is a CDK9 inhibitor (IC_50_ of <3 nM), highly selective (>10 fold) against all other CDKs and kinases. ➢ Induces a rapid apoptosis in broadly across hematologic cancer models in vitro *and* in vivo*, with a* minimal effect on solid tumors. ➢ Causes a rapid dose- and time-dependent decrease in RNAPII Ser2 phosphorylation with loss of Mcl-1 and MYC mRNA and protein, resulting in caspase activation and a reduced cell viability. In contrast, Bcl2 and Bclx_L_ remained unchanged ➢ Is an effective short half-life treatment as single agent or in combination, for patients with hematological malignancies.	33306391, 31699827 *Abstract. Cancer Res* (2018) 78 (13_Sup-plement): 310
**CCNT1**	**CycT1is** C3	➢ Derivative of a hit found from *in* *silico* screening of small molecules that bind to the CycT1/Tat/TAR interaction interface. ➢ Prevents Tat-CCNT1 binding thus Tat-mediated transcription. ➢ Suppresses acute viral replication and HIV-1 reactivation from latency cell lines with minimal cytotoxicity. IC_50s_ varies from: 9.6-617 nM and CC_50_ from >1000 to >10,000. ➢ Not yet characterized in in vivo models of HIV-1 latency.	23274668 30351168

**Table 2:** Other host factors inhibitors (part 1) and (part 2).

OTHERS HOST INHIBITORS		MECHANISM OF ACTION	PMID
**P300**	**LTK14**	➢ Derivative of the natural product garcinol. ➢ Selective inhibitor of histone acetyltransferase p300 (5-7µM). ➢ Inhibit acute infection in a CD4+ T cell line at high µM concentrations, without toxicity (>50 µM). ➢ No evidence in latency models.	17584612, 30351168
**NFAT**	**Cyclosporin A** (CSA)	➢ Inhibits NFAT-mediated HIV-1 transcription in primary CD4+ T cells. ➢ Prevents the dephosphorylation of NFAT, which is essential for NFAT’s nuclear translocation and activation, resulting in disruption of T cell activation. ➢ Suppresses proliferation of cytotoxic T cells and inhibits the production of T cell-derived mediators such as interleukin-2 (IL-2). ➢ Used in conjunction with ART as an immune-modulatory agent in clinical trials, with low toxicity. ➢ Inhibits Ca^2+^/calmodulin-dependent protein phosphatase (IC_50_ of 65 nM), cholecystokinin-(100 pM), or carbamylcholine- (10 µM), induced amylase release. ➢ Nephrotoxic effects.	10692237, 30351168, 7515049, 7542793
	**Fujimycine** FK506	➢ Calcium dependent protein phosphatase calcineurin, responsible for the dephosphorylation of NFAT. ➢ Inhibits antigen and mitogen triggered T cell activation. ➢ Up to 100-fold more potent than CSA in various models. ➢ Partially inhibits the Ca2+/calmodulin-dependent protein phosphatase activity but did not significantly inhibit amylase secretion at concentrations up to 1 µM. ➢ Nephrotoxic effects.	7542793, 1381509, 30351168
**NF-kB**	**ACHP**	➢ Inhibits IκB kinase β (IKKβ, IC_50_ = 8.5 nM ) and IKKα (IC_50_ = 250 nM). ➢ Reduces the constitutive phosphorylation of IκBα and NF-κB p65 in myeloma cells at 50 µM. ➢ Prevents HIV-1 reactivation induced by TNF-α in HIV latently infected cells (IC_50_ = 0.56 µM).	15225717, 16436709, 12617920,
	**Noraris-teromycin**	➢ Inhibits IKKα and weakly IKKβ phosphorylation and degradation upon cellular reactivation with TNF-α treatment. ➢ Prevents p65 phosphorylation. ➢ Suppresses HIV-1 viral replication upon cellular reactivation with TNF-α treatment of HIV cell models of latency (OM10.1 and Molt4/IIIB, IC_50_~100 nM, CC_50_ > 10 µM).	18713798
**mTOR**	**PP242** Torkinib	➢ Competes with ATP for its binding site and inhibits both mTORC1 and mTORC2 with an IC_50_ of 8 nM in in vitro assays; with >10- and 100-fold selectivity for mTOR than PI3Kδ or PI3Kα/β/γ, respectively. ➢ Suppresses HIV reactivation of latent HIV upon T-cell stimulants both in the Bcl-2 HIV latency primary cell model and in CD4+ T cells from HIV suppressed individuals under ART, without affecting cellular viability. ➢ Abrogated Tat-independent and -dependent transactivation of the HIV promoter; and at doses (200 nM–1000 nM) reduces CDK9 phosphorylation in CD3/CD28-stimulated CD4+ T cells from uninfected donors.	27978436, 19209957, 18849971, 30351168
	**Torin**	➢ Competes with ATP and inhibits both mTORC1 and mTORC2, with IC_50_ values between 2 and 10 nM. Exhibits 1000-fold selectivity for mTOR over PI3K (IC_50_: 1800 nM) and 100-fold binding selectivity relative to 450 other kinases. ➢ Suppresses HIV reactivation of latent HIV upon T-cell stimulants both in the Bcl-2 HIV latency primary cell model and in CD4+ T cells from HIV suppressed individuals under ART, without affecting cellular viability. ➢ Abrogated Tat-independent and -dependent transactivation of the HIV promoter.	27978436, 20860370, 21651476, 22125084, 30351168
	**Rapamycin** AY-22989, Rapamune, Sirolimus, NSC-2260804	➢ Forms a complex with FKBP12 and binds to mTORC1 causing its inhibition. mTORC2 is insensitive to Rapamycin. ➢ Suppresses HIV reactivation upon T-cell stimulants in the Bcl-2 HIV latency primary cell model, but with less potency than Torin and PP242, and a a slight decrease of viability. ➢ Abrogated Tat-independent transactivation of the HIV promoter in a dose-dependent manner.	27978436, 17350953
**TFIIH**	**Spironolactone (SP)** SC9420	➢ Aldosterone antagonist approved for clinical use, degrades the XPB cellular helicase, a TFIIH component. ➢ Inhibits HIV-1 and HIV-2 infection of permissive T cells. ➢ Blocks Tat-dependent transactivation of the HIV promoter. ➢ Inhibits HIV-1 replication and reactivation in HIV latent cell models, primary CD4+ T cells, and HIV suppressed individuals under ART, with variable toxicity levels. ➢ Reduces RNAPII recruitment to the HIV-1 genome. ➢ Long-term treatment with SP does not result in epigenetic suppression of HIV since HIV rebounds upon SP treatment interruption. ➢ Long-term treatment with SP does not lead to significant global dysregulation of cellular transcripts.	28842263, 30351168, 27681137, 33239456, 32573496
**PI3K/Akt** pathway	**BPRHIV001**	➢ Represses the phosphorylation of PDPK1, resulting in the repression of the phosphorylation of Akt. Akt is then not able to protect p300 from degradation. P300 known to modulate Tat function through acetylation, its decrease results in subsequent inhibition of HIV-1 Tat-Mediated Transcription (IC_50_: 1.3 nM in HeK293T cells). ➢ No evidence in latency models or in vivo.	21697490
**FACT**	**Curaxin** CBL0100	➢ Inhibits acute HIV-1 replication in a CD4+ T cell line (IC_50_ = 0.055 μM, CC_50_ > 0.2 µM) and PBMCs. ➢ Reduces HIV-1 reactivation in latency cell lines and primary CD4+ T cell model of HIV-1 latency. ➢ Suppresses HIV-1 transcriptional elongation by reducing the HIV promoter occupancy of RNAP II and FACT. ➢ Proposed mechanism: HIV-1 Tat associates with FACT recruits it in the proximity to nuc-1. FACT facilitates the disassembly/reassembly of the nuc-1 to allow the RNAP II transcriptional elongation. CBL0100 intercalates into chromatins and blocks the FACT accessibility/association with nuc-1, preventing the subsequent steps.	29089933, 30351168
**BRD4**	**ZL0580**	➢ Structurally close analog to ZL0590. Suppresses HIV by selectively binding to BD1 domain of BRD4. Mechanistically different from the BET/BRD4 pan-inhibitor JQ1, which non selectively binds to BD1 and BD2 domains of all BET proteins. ➢ Suppresses acute and latent HIV replication and reactivation at micromolar range in in vitro and ex vivo HIV cell models. ➢ Inhibits transcription elongation and induces a repressive chromatin environment at the HIV promoter. ➢ PBMCs of aviremic HIV-infected individuals treated both with ART and ZL0580 accelerated HIV suppression during ART and delayed viral rebound after ART cessation.	31329163, 31936859, 31733396
**JAK/STAT**	**Ruxolitinib** INCB018424	➢ FDA approved for rheumatoid arthritis and a potent and selective ATP-competitive inhibitor of JAK 1, 2, and 3 with an IC_50_ value of 2.7, 4.5, and 322 nM, respectively. ➢ Sub micromolar inhibition of HIV-1, HIV-2, and SIV, RT-SHIV, across primary human or rhesus macaque lymphocytes and macrophages, with no significant cytotoxicity at 2 to 3 logs above their effective antiviral concentration. ➢ Inhibits reactivation of latent HIV at low-micromolar concentrations across the J-Lat T cell latency models and in primary human central memory lymphocytes, with variable toxicity levels. ➢ Decreases the frequency of cells harboring HIV integrated DNA in cultures of T cells activated by TCR in the presence or absence of ART with doses of Ruxolitinib as low as 0.01 μM. ➢ Significantly blocks IL-2, IL-7, and IL-15 induced HIV reactivation upon γ-C cytokines stimulation in in vitro and ex vivo CD4 T cell cultures. ➢ Clinical trial phase 2 using 10 mg of Ruxolitinib twice daily in combination with ART for 5 weeks vs ART alone, in, aviremic HIV-infected individuals showed well-tolerated treatment, no significant reduction of IL-6 and decrease of markers of immune activation known to be associated with poor HIV outcomes.	33693561, 24419350, 29267399, 22422826, 31936859, 32573496
	**Tofacitinib** CP-690550, Tasocitinib, Xeljanz	➢ FDA approved drug for myelofibrosis and JAK3 and 1 inhibitor. ➢ Potent inhibitor of inflammatory cytokines with resultant immunosuppressive and anti-inflammatory activity. ➢ Very similar activities to Ruxolitinib: with 1) submicromolar inhibition of infection with HIV-1, HIV-2, and SIV, RT-SHIV, across primary human or rhesus macaque lymphocytes and macrophages, with no significant cytotoxicity at 2 to 3 logs above their effective antiviral concentration; 2) inhibited reactivation of latent HIV-1 at low-micromolar concentrations across the J-Lat T cell latency models and in primary human central memory lymphocytes, with variable toxicity levels; 3) decreases the frequency of cells harboring HIV integrated DNA in cultures of T cells activated by TCR in the presence or absence of ART; 4) significantly blocks IL-2, IL-7, and IL-15 induced HIV reactivation upon γ-C cytokines stimulation in in vitro and ex vivo CD4+ T cell cultures.	24419350, 29267399, 31936859
	**Filgotinib** GLPG0634	➢ Selective JAK inhibitor with IC_50_ of 10 nM, 28 nM, 810 nM, and 116 nM for JAK1, JAK2, JAK3, and TYK2. ➢ Suppresses HIV replication in CD4^+^ T cells from aviremic HIV-infected individuals and cell lines at µM range. ➢ Suppresses HIV-1 splicing mRNA while Ruxolitinib reduces unspliced mRNAs. ➢ Reduces T cell activation from virally suppressed ART treated individuals. ➢ Suppresses HIV driven aberrant cancer-related gene expression at the integration site in cell line. ➢ Significantly reduces the frequency of cells harboring inducible HIV in a T cell line. ➢ Transcriptome analysis revealed that Filgotinib suppresses T cell activation *via* the JAK/STAT signaling pathway, alters RNA processing and chromatin organization.	32573496
**PP1**	**1H4**	➢ Targets the “RVxF”-binding cavity of PP1 to disrupt the interaction of PP1 with Tat and inhibit HIV replication. ➢ Inhibits HIV transcription and replication with IC_50_ 10 µM, CC_50_ > 25 µM in a T cell line. ➢ Prevents the translocation of PP1 to the nucleus.	22768081
	**1E7-03** Compound 7C	➢ Acts like 1H4 but with an IC_50_ 5-fold lower than 1H4, with no toxicity and a plasma half-life greater than 8 h in mice. ➢ Enhances trans-endothelial migration of HIV-Tg macrophages in vitro*,* decreased lung neutrophil infiltration in vivo, and increases lung macrophage levels in HIV-Tg mice. Moreover, it reduces levels of inflammatory IL-6 cytokine, improves bleeding score and decreases lung injury.	25073485

#### 3.2.2. NF-κB Inhibitors

NF-κB has been widely explored for HIV transcriptional regulation [199,200,201,202,203]. The activation of NF-κB is rapid and occurs within minutes after exposure to a relevant inducer, does not require de novo protein synthesis and prompts the strong transcriptional activation of specific viral and cellular genes [204]. Three NF-kB signaling pathways have been described in the literature. The canonical pathway is triggered by numerous signals, including those mediated by innate and adaptive immune receptors. It requires the activation of the IKK complex by Tak1, IKK-mediated IκBα phosphorylation, ubiquitination and the degradation of IκBα, leading to the nuclear translocation of the NF-κB heterodimer p65/p50 and target gene activation [205]. The non-canonical NF-κB pathway involves the phosphorylation-induced p100 processing and is initiated by signaling from TNFR members. This pathway relies on NIK and IKKα, but not on the IKK complex, and activates the hetero-dimer RelB/p52 [205]. The atypical NF-kB signaling pathway is triggered by genotoxic stress upon the activation of the NF-κB essential modulator (NEMO). The agonist activation of this pathway results in the phosphorylation of the p105 by the IKK complex, p105 polyubiquitination and degradation and the nuclear translocation of p50 homodimers to regulate target gene transcription [206]. Therefore, NF-κB inhibitors can be grouped into multiple categories based on the NF-κB signaling pathways (Table 2).

##### IKK Inhibitors

These inhibitors block IκB Phosphorylation and degradation, necessary for NF-κB Release. The few IKK inhibitors reported can be classified into three groups: ATP analogs that specifically interact with IKK (such as SC-839); molecules that allosterically impact IKK structure (such as BMS-345541); or compounds interacting with a specific cysteine residue in the activation loop of the IKKβ subunit (such as parthenolide, arsenite) [207].

##### Proteasome Inhibitors and IκB Ubiquitination Blockers

Very potent proteasome inhibitors have been identified, such as lactacystine, MG132 and salinosporamide A (NPI-0052) [208]. The small molecule R0196-9920 has been reported to specifically inhibit IκBα ubiquitination in mouse models [209].

##### NF-κB Nuclear Translocation Inhibitors

SN50, a forty-one-residue synthetic peptide containing a hydrophobic membrane-translocating region and the nuclear localization sequence of the p50 subunit of NF-κB, can cross cell membranes and compete with the nuclear translocation of NF-κB [210].

##### p65 Acetylation Inhibitors

The acetylation of the activated p65 subunit of NF-κB in the nucleus increases its DNA-binding affinity. Several compounds inhibiting acetylation have been reported to block NF-κB activation. For instance, natural gallic acid [211] and anacardic acid derived from traditional plants [212].

##### NF-κB-DNA Binding Inhibitors

This is the most direct strategy to block NF-κB binding to DNA. Some sesquiterpene lactones have been reported to inhibit NF-κB by interacting with the residue, cysteine 38, in the DNA-binding loop of p65 [213], while some decoy oligodeoxynucleotides (ODNs) have κB binding sites and compete for NF-κB dimer binding to specific genomic promoters [214].

##### Antioxidant Inhibitors

Oxidative stress was reported to activate the NF-κB signaling pathway [215], thus antioxidant inhibitors were also studied as possible NF-κB inhibitors [216,217]. These include mitochondrial electron transport inhibitors (e.g., rotenone) [218], antioxidizing enzymes (e.g., manganese superoxide dismutase and catalase) [219], N-acetyl-L-cysteine and calcium chelators. Interestingly, the mechanism of certain antioxidant inhibitors does not fully correlate with their antioxidant properties but with a more selective and specific mechanism to NF-κB. For instance, N-acetyl-L-cysteine selectively blocks TNF-induced signaling by reducing the affinity of the receptor to TNF or the pyrrolidine dithiocarbamate that inhibits the IκB ubiquitin ligase activity in a cell-free system [220].

##### Inhibitors Targeting Related Signaling Pathways or Molecules That Affect NF-κB Activation

This class of inhibitors includes PKC inhibitors (golli BG21), NIK inhibitors (betaine) or HSP90 inhibitors (17-AAG and AUY922) [201].

Despite extensive work that went into investigating these NF-KB signaling pathways to block or reactivate the latent virus from reservoirs in PLWH, the fine balance between the activity and the safety of these inhibitors has yet to be optimized.

#### 3.2.3. TFIIH Inhibitors

TFIIH is a ten-protein complex consisting of a core (XPB, XPD, p62, p52, p44, p34 and p8) and a CDK-activating kinase (CAK) subcomplex (CDK7, cyclin H and MAT1) [221]. It plays a critical role in facilitating transcription initiation by opening the DNA strands around the transcription start site and the phosphorylation of the C-terminal domain of RNAPII for activation [222]. The neutralization of some of the TFIIH subunits, such as XPB, did not impact cellular homeostasis [124]; thus, TFIIH subunits have become a new target for drug discovery. The FDA-approved mineralocorticoid receptor (MR) antagonist spironolactone (SP) is used to treat a variety of disparate conditions ranging from heart failure to high blood pressure [223]. Interestingly, recent in vitro cell-based drug repurposing screens have identified SP as a compound with additional functions such as inhibiting DNA repair [224] and viral infection [225]. SP acts by rapidly degrading XPB protein [226], and it was shown that SP inhibits acute HIV and HIV-2 transcription without affecting cell viability in cell lines and primary CD4^+^ T cells [227]. SP was also shown to inhibit HIV reactivation from latency at the micromolar range in both cell line models and resting CD4^+^ T cells isolated from aviremic-infected individuals [124]. This activity of SP correlates with a reduction in RNAPII recruitment to the HIV promoter and is independent of the Tat-TAR axis. Unlike dCA, the long-term pre-treatment of chronically infected cells with SP did not result in the sustained epigenetic suppression of HIV, since upon SP treatment, the interruption virus rapidly rebound Importantly, the long-term degradation of XPB does not affect cellular transcriptomics. Further studies are, however, needed to determine the mechanisms behind the increased susceptibility of HIV to SP treatment compared to the host machinery.

Other HIV transcriptional inhibitors are currently in development, such as CDK2, NFAT, HATs, HDMs, P300/CBP and mTOR inhibitors and can be found in Table 2.

## 4. Conclusions

The last decade has brought forward important research exploring HIV functional cure strategies. Many remarkable technologies and discoveries helped shape its development, namely genome editing, immune modulators, recombinant antibody therapy, novel small molecules and gene targets discovered through numerous screens. The identification of latently infected cells from the uninfected cells remains a major hurdle, and additional research is needed to identify the phenotypic markers of latently infected cells, which can be used in targeted therapeutic approaches.

With this review, we provided a comprehensive compilation of transcriptional inhibitors that may be used as tools to further our understanding of the transcriptional regulation of HIV or as groundwork for drug development. From the sole perspective of efficacy and toxicity, so far, few of these transcriptional inhibitors have shown therapeutic potential against HIV. In this category, we can include the Tat inhibitor dCA that is currently in preclinical tests, the FDA-approved drug Spironolactone which promotes the degradation of the XPB subunit of TFIIH, the C3 cyclin T1 inhibitor that binds the interface of HIV Tat bound to cyclin T1 or, lastly, the inhibitor of the FACT complex, Curaxin. Interestingly, FACT is typically expressed during development or in various tumor cells and is associated with tumor aggressiveness and poor prognosis [228], while mostly absent in healthy cells. Its expression in CD4^+^ T cells may thus be directly dependent on HIV infection and could be explored as an HIV biomarker. HIV-associated comorbidities would benefit from the CDK9 inhibitors, AZD4573 and Voruciclib, tested in clinical trials in individuals with malignancies (NCT05140382 and NCT03547115, respectively), while immune activation associated with poor HIV prognosis in the presence of ART could be reverted with JAK/STAT inhibitors, namely Ruxolitinib, which seems well-tolerated in PLWH on ART and is in clinical trial phase II [229].

Once successful candidates are identified, many questions still need to be addressed as to the duration and accessibility of a cure for PLWH. Namely, what would be the duration and frequency of the transcriptional inhibitor’s treatment to fully inhibit residual viral production in PLWH? What is the interval of treatment interruption before the viral rebound, if any, is observed? What is considered a successful time frame of viral suppression without ART? Would transcriptional inhibitors be needed in the absence of ART to maintain undetectable viral production? Would they be beneficial in front-line therapy to reduce the size of the established reservoir? Would these inhibitors be prone to the evolution of viral resistance? Finally, would they be able to soothe HIV-associated diseases?

The combination of multiple approaches, such as the “shock-and-kill” with the “block-and-lock”, may likely improve outcomes. Easily reactivated viruses could first be “flushed” out with the shock-and-kill approach; latency-promoting agents would then silence the remaining proviruses. Certainly, rigorous evaluation and validation of this combined approach in vitro and in vivo will be needed, but supported by hopeful work on this front, we remain optimistic.

## References

[1] Marconi V.C., Moser C., Gavegnano C., Deeks S.G., Lederman M.M., Overton E.T., Tsibris A., Hunt P.W., Kantor A., Sekaly R.P. (2022). Randomized Trial of Ruxolitinib in Antiretroviral-Treated Adults with Human Immunodeficiency Virus. Clin. Infect. Dis..

[2] Llibre J.M., Hung C.C., Brinson C., Castelli F., Girard P.M., Kahl L.P., Blair E.A., Angelis K., Wynne B., Vandermeulen K. (2018). Efficacy, safety, and tolerability of dolutegravir-rilpivirine for the maintenance of virological suppression in adults with HIV-1: Phase 3, randomised, non-inferiority SWORD-1 and SWORD-2 studies. Lancet.

[3] Gupta R.K., Abdul-Jawad S., McCoy L.E., Mok H.P., Peppa D., Salgado M., Martinez-Picado J., Nijhuis M., Wensing A.M.J., Lee H. (2019). HIV-1 remission following CCR5Δ32/Δ32 haematopoietic stem-cell transplantation. Nature.

[4] Gupta R.K., Peppa D., Hill A.L., Gálvez C., Salgado M., Pace M., McCoy L.E., Griffith S.A., Thornhill J., Alrubayyi A. (2020). Evidence for HIV-1 cure after CCR5Δ32/Δ32 allogeneic haemopoietic stem-cell transplantation 30 months post analytical treatment interruption: A case report. Lancet HIV.

[5] The Lancet Hiv (2019). Like London buses, two putative cure cases arrive at once. Lancet HIV.

[6] McNicholl J.M., Smith D.K., Qari S.H., Hodge T. (1997). Host genes and HIV: The role of the chemokine receptor gene CCR5 and its allele. Emerg. Infect. Dis..

[7] Barouch D.H., Deeks S.G. (2014). Immunologic strategies for HIV-1 remission and eradication. Science.

[8] Henderson L.J., Reoma L.B., Kovacs J.A., Nath A. (2020). Advances toward Curing HIV-1 Infection in Tissue Reservoirs. J. Virol..

[9] Sadowski I., Hashemi F.B. (2019). Strategies to eradicate HIV from infected patients: Elimination of latent provirus reservoirs. Cell. Mol. Life Sci..

[10] Xu W., Li H., Wang Q., Hua C., Zhang H., Li W., Jiang S., Lu L. (2017). Advancements in Developing Strategies for Sterilizing and Functional HIV Cures. Biomed Res. Int..

[11] Siliciano J.D., Kajdas J., Finzi D., Quinn T.C., Chadwick K., Margolick J.B., Kovacs C., Gange S.J., Siliciano R.F. (2003). Long-term follow-up studies confirm the stability of the latent reservoir for HIV-1 in resting CD4+ T cells. Nat. Med..

[12] Chun T.W., Engel D., Berrey M.M., Shea T., Corey L., Fauci A.S. (1998). Early establishment of a pool of latently infected, resting CD4(+) T cells during primary HIV-1 infection. Proc. Natl. Acad. Sci. USA.

[13] Mzingwane M.L., Tiemessen C.T. (2017). Mechanisms of HIV persistence in HIV reservoirs. Rev. Med. Virol..

[14] Ramskold D., Wang E.T., Burge C.B., Sandberg R. (2009). An abundance of ubiquitously expressed genes revealed by tissue transcriptome sequence data. PLoS Comput. Biol..

[15] Rands C.M., Meader S., Ponting C.P., Lunter G. (2014). 8.2% of the Human genome is constrained: Variation in rates of turnover across functional element classes in the human lineage. PLoS Genet..

[16] Jiang C., Lian X., Gao C., Sun X., Einkauf K.B., Chevalier J.M., Chen S.M.Y., Hua S., Rhee B., Chang K. (2020). Distinct viral reservoirs in individuals with spontaneous control of HIV-1. Nature.

[17] Kessing C.F., Nixon C.C., Li C., Tsai P., Takata H., Mousseau G., Ho P.T., Honeycutt J.B., Fallahi M., Trautmann L. (2017). In Vivo Suppression of HIV Rebound by Didehydro-Cortistatin A, a “Block-and-Lock” Strategy for HIV-1 Treatment. Cell Rep..

[18] Li C., Mousseau G., Valente S.T. (2019). Tat inhibition by didehydro-Cortistatin A promotes heterochromatin formation at the HIV-1 long terminal repeat. Epigenetics Chromatin.

[19] Mori L., Jenike K., Yeh Y.J., Lacombe B., Li C., Getzler A., Mediouni S., Cameron M., Pipkin M., Ho Y.C. (2020). The XPB Subunit of the TFIIH Complex Plays a Critical Role in HIV-1 Transcription and XPB Inhibition by Spironolactone Prevents HIV-1 Reactivation from Latency. J. Virol..

[20] Mousseau G., Clementz M.A., Bakeman W.N., Nagarsheth N., Cameron M., Shi J., Baran P., Fromentin R., Chomont N., Valente S.T. (2012). An analog of the natural steroidal alkaloid cortistatin A potently suppresses Tat-dependent HIV transcription. Cell. Host. Microbe.

[21] Vansant G., Bruggemans A., Janssens J., Debyser Z. (2020). Block-And-Lock Strategies to Cure HIV Infection. Viruses.

[22] Li C., Mori L., Valente S.T. (2021). The Block-and-Lock Strategy for Human Immunodeficiency Virus Cure: Lessons Learned from Didehydro-Cortistatin A. J. Infect. Dis..

[23] Perkins M.J., Bradley W.P., Lalani T., Agan B.K., Whitman T.J., Ferguson T.M., Okulicz J.F., Ganesan A. (2017). Brief Report: Prevalence of Posttreatment Controller Phenotype Is Rare in HIV-Infected Persons After Stopping Antiretroviral Therapy. J. Acquir. Immune Defic. Syndr..

[24] Schiralli Lester G.M., Henderson A.J. (2012). Mechanisms of HIV Transcriptional Regulation and Their Contribution to Latency. Mol. Biol. Int..

[25] Richman D.D., Margolis D.M., Delaney M., Greene W.C., Hazuda D., Pomerantz R.J. (2009). The challenge of finding a cure for HIV infection. Science.

[26] Valente S.T. (2020). Key Players in HIV-1 Transcriptional Regulation: Targets for a Functional Cure. Viruses.

[27] Sainsbury S., Bernecky C., Cramer P. (2015). Structural basis of transcription initiation by RNA polymerase II. Nat. Rev. Mol. Cell. Biol..

[28] Harlen K.M., Churchman L.S. (2017). The code and beyond: Transcription regulation by the RNA polymerase II carboxy-terminal domain. Nat. Rev. Mol. Cell. Biol..

[29] Dutilleul A., Rodari A., Van Lint C. (2020). Depicting HIV-1 Transcriptional Mechanisms: A Summary of What We Know. Viruses.

[30] Barboric M., Nissen R.M., Kanazawa S., Jabrane-Ferrat N., Peterlin B.M. (2001). NF-kappaB binds P-TEFb to stimulate transcriptional elongation by RNA polymerase II. Mol. Cell..

[31] Van Lint C., Bouchat S., Marcello A. (2013). HIV-1 transcription and latency: An update. Retrovirology.

[32] Tripathy M.K., Abbas W., Herbein G. (2011). Epigenetic regulation of HIV-1 transcription. Epigenomics.

[33] Agosto L.M., Gagne M., Henderson A.J. (2015). Impact of Chromatin on HIV Replication. Genes.

[34] Kaczmarek K., Morales A., Henderson A.J. (2013). T Cell Transcription Factors and Their Impact on HIV Expression. Virology.

[35] Morton E.L., Forst C.V., Zheng Y., DePaula-Silva A.B., Ramirez N.P., Planelles V., D’Orso I. (2019). Transcriptional Circuit Fragility Influences HIV Proviral Fate. Cell Rep..

[36] Bacher S., Meier-Soelch J., Kracht M., Schmitz M.L. (2021). Regulation of Transcription Factor NF-kappaB in Its Natural Habitat: The Nucleus. Cells.

[37] Wu T., Kamikawa Y.F., Donohoe M.E. (2018). Brd4’s Bromodomains Mediate Histone H3 Acetylation and Chromatin Remodeling in Pluripotent Cells through P300 and Brg1. Cell Rep..

[38] Devaiah B.N., Lewis B.A., Cherman N., Hewitt M.C., Albrecht B.K., Robey P.G., Ozato K., Sims R.J., Singer D.S. (2012). BRD4 is an atypical kinase that phosphorylates serine2 of the RNA polymerase II carboxy-terminal domain. Proc. Natl. Acad. Sci. USA.

[39] Kiernan R.E., Vanhulle C., Schiltz L., Adam E., Xiao H., Maudoux F., Calomme C., Burny A., Nakatani Y., Jeang K.T. (1999). HIV-1 tat transcriptional activity is regulated by acetylation. EMBO J..

[40] Luo Z., Lin C., Guest E., Garrett A.S., Mohaghegh N., Swanson S., Marshall S., Florens L., Washburn M.P., Shilatifard A. (2012). The super elongation complex family of RNA polymerase II elongation factors: Gene target specificity and transcriptional output. Mol. Cell. Biol..

[41] Tang D., Chen C., Liao G., Liu J., Liao B., Huang Q., Chen Q., Zhao J., Jiang H., Duan J. (2020). Structural and functional insight into the effect of AFF4 dimerization on activation of HIV-1 proviral transcription. Cell Discov..

[42] Wu J., Xue Y., Gao X., Zhou Q. (2020). Host cell factors stimulate HIV-1 transcription by antagonizing substrate-binding function of Siah1 ubiquitin ligase to stabilize transcription elongation factor ELL2. Nucleic Acids Res..

[43] Easley R., Carpio L., Dannenberg L., Choi S., Alani D., Van Duyne R., Guendel I., Klase Z., Agbottah E., Kehn-Hall K. (2010). Transcription through the HIV-1 nucleosomes: Effects of the PBAF complex in Tat activated transcription. Virology.

[44] Marzio G., Tyagi M., Gutierrez M.I., Giacca M. (1998). HIV-1 tat transactivator recruits p300 and CREB-binding protein histone acetyltransferases to the viral promoter. Proc. Natl. Acad. Sci. USA.

[45] Mahmoudi T. (2012). The BAF complex and HIV latency. Transcription.

[46] Rafati H., Parra M., Hakre S., Moshkin Y., Verdin E., Mahmoudi T. (2011). Repressive LTR nucleosome positioning by the BAF complex is required for HIV latency. PLoS Biol..

[47] Pagans S., Pedal A., North B.J., Kaehlcke K., Marshall B.L., Dorr A., Hetzer-Egger C., Henklein P., Frye R., McBurney M.W. (2005). SIRT1 regulates HIV transcription via Tat deacetylation. PLoS Biol..

[48] Karn J. (2011). The molecular biology of HIV latency: Breaking and restoring the Tat-dependent transcriptional circuit. Curr. Opin. HIV AIDS.

[49] Boehm D., Ott M. (2017). Host Methyltransferases and Demethylases: Potential New Epigenetic Targets for HIV Cure Strategies and Beyond. AIDS Res. Hum. Retrovir..

[50] Dahabieh M.S., Battivelli E., Verdin E. (2015). Understanding HIV latency: The road to an HIV cure. Annu. Rev. Med..

[51] Mbonye U., Karn J. (2014). Transcriptional control of HIV latency: Cellular signaling pathways, epigenetics, happenstance and the hope for a cure. Virology.

[52] Margolis D.M., Archin N.M., Cohen M.S., Eron J.J., Ferrari G., Garcia J.V., Gay C.L., Goonetilleke N., Joseph S.B., Swanstrom R. (2020). Curing HIV: Seeking to Target and Clear Persistent Infection. Cell.

[53] Sengupta S., Siliciano R.F. (2018). Targeting the Latent Reservoir for HIV-1. Immunity.

[54] Marino-Ramirez L., Kann M.G., Shoemaker B.A., Landsman D. (2005). Histone structure and nucleosome stability. Expert Rev. Proteom..

[55] Verdin E. (1991). DNase I-hypersensitive sites are associated with both long terminal repeats and with the intragenic enhancer of integrated human immunodeficiency virus type 1. J. Virol..

[56] Saha A., Wittmeyer J., Cairns B.R. (2006). Chromatin remodelling: The industrial revolution of DNA around histones. Nat. Rev. Mol. Cell. Biol..

[57] Conrad R.J., Fozouni P., Thomas S., Sy H., Zhang Q., Zhou M.M., Ott M. (2017). The Short Isoform of BRD4 Promotes HIV-1 Latency by Engaging Repressive SWI/SNF Chromatin-Remodeling Complexes. Mol. Cell.

[58] Tomar S., Ali I., Ott M. (2018). A BAF’ling Approach to Curing HIV. Cell Chem. Biol..

[59] Stoszko M., De Crignis E., Rokx C., Khalid M.M., Lungu C., Palstra R.J., Kan T.W., Boucher C., Verbon A., Dykhuizen E.C. (2016). Small Molecule Inhibitors of BAF. A Promising Family of Compounds in HIV-1 Latency Reversal. eBioMedicine.

[60] Mahmoudi T., Parra M., Vries R.G., Kauder S.E., Verrijzer C.P., Ott M., Verdin E. (2006). The SWI/SNF chromatin-remodeling complex is a cofactor for Tat transactivation of the HIV promoter. J. Biol. Chem..

[61] Tréand C., du Chéné I., Brès V., Kiernan R., Benarous R., Benkirane M., Emiliani S. (2006). Requirement for SWI/SNF chromatin-remodeling complex in Tat-mediated activation of the HIV-1 promoter. EMBO J..

[62] Mizutani T., Ishizaka A., Tomizawa M., Okazaki T., Yamamichi N., Kawana-Tachikawa A., Iwamoto A., Iba H. (2009). Loss of the Brm-type SWI/SNF chromatin remodeling complex is a strong barrier to the Tat-independent transcriptional elongation of human immunodeficiency virus type 1 transcripts. J. Virol..

[63] Marian C.A., Stoszko M., Wang L., Leighty M.W., de Crignis E., Maschinot C.A., Gatchalian J., Carter B.C., Chowdhury B., Hargreaves D.C. (2018). Small Molecule Targeting of Specific BAF (mSWI/SNF) Complexes for HIV Latency Reversal. Cell Chem. Biol..

[64] Gallastegui E., Millán-Zambrano G., Terme J.M., Chávez S., Jordan A. (2011). Chromatin reassembly factors are involved in transcriptional interference promoting HIV latency. J. Virol..

[65] Rodgers M.J., Banks D.J., Bradley K.A., Young J.A. (2014). CHD1 and CHD2 are positive regulators of HIV-1 gene expression. Virol. J..

[66] Tan M., Luo H., Lee S., Jin F., Yang J.S., Montellier E., Buchou T., Cheng Z., Rousseaux S., Rajagopal N. (2011). Identification of 67 histone marks and histone lysine crotonylation as a new type of histone modification. Cell.

[67] Strahl B.D., Allis C.D. (2000). The language of covalent histone modifications. Nature.

[68] Gregoretti I.V., Lee Y.M., Goodson H.V. (2004). Molecular evolution of the histone deacetylase family: Functional implications of phylogenetic analysis. J. Mol. Biol..

[69] Romerio F., Gabriel M.N., Margolis D.M. (1997). Repression of human immunodeficiency virus type 1 through the novel cooperation of human factors YY1 and LSF. J. Virol..

[70] Williams S.A., Chen L.F., Kwon H., Ruiz-Jarabo C.M., Verdin E., Greene W.C. (2006). NF-kappaB p50 promotes HIV latency through HDAC recruitment and repression of transcriptional initiation. EMBO J..

[71] Keedy K.S., Archin N.M., Gates A.T., Espeseth A., Hazuda D.J., Margolis D.M. (2009). A limited group of class I histone deacetylases acts to repress human immunodeficiency virus type 1 expression. J. Virol..

[72] Huber K., Doyon G., Plaks J., Fyne E., Mellors J.W., Sluis-Cremer N. (2011). Inhibitors of histone deacetylases: Correlation between isoform specificity and reactivation of HIV type 1 (HIV-1) from latently infected cells. J. Biol. Chem..

[73] Tyagi M., Karn J. (2007). CBF-1 promotes transcriptional silencing during the establishment of HIV-1 latency. EMBO J..

[74] Li Z., Mbonye U., Feng Z., Wang X., Gao X., Karn J., Zhou Q. (2018). The KAT5-Acetyl-Histone4-Brd4 axis silences HIV-1 transcription and promotes viral latency. PLoS Pathog..

[75] Greer E.L., Shi Y. (2012). Histone methylation: A dynamic mark in health, disease and inheritance. Nat. Rev. Genet..

[76] Friedman J., Cho W.K., Chu C.K., Keedy K.S., Archin N.M., Margolis D.M., Karn J. (2011). Epigenetic silencing of HIV-1 by the histone H3 lysine 27 methyltransferase enhancer of Zeste 2. J. Virol..

[77] Imai K., Togami H., Okamoto T. (2010). Involvement of histone H3 lysine 9 (H3K9) methyltransferase G9a in the maintenance of HIV-1 latency and its reactivation by BIX01294. J. Biol. Chem..

[78] Ding D., Qu X., Li L., Zhou X., Liu S., Lin S., Wang P., Liu S., Kong C., Wang X. (2013). Involvement of histone methyltransferase GLP in HIV-1 latency through catalysis of H3K9 dimethylation. Virology.

[79] Nguyen K., Das B., Dobrowolski C., Karn J. (2017). Multiple Histone Lysine Methyltransferases Are Required for the Establishment and Maintenance of HIV-1 Latency. mBio.

[80] Zhang T., Cooper S., Brockdorff N. (2015). The interplay of histone modifications—writers that read. EMBO Rep..

[81] Boehm D., Jeng M., Camus G., Gramatica A., Schwarzer R., Johnson J.R., Hull P.A., Montano M., Sakane N., Pagans S. (2017). SMYD2-Mediated Histone Methylation Contributes to HIV-1 Latency. Cell Host Microbe.

[82] Zhang Z., Nikolai B.C., Gates L.A., Jung S.Y., Siwak E.B., He B., Rice A.P., O’Malley B.W., Feng Q. (2017). Crosstalk between histone modifications indicates that inhibition of arginine methyltransferase CARM1 activity reverses HIV latency. Nucleic Acids Res..

[83] Jiang G., Nguyen D., Archin N.M., Yukl S.A., Méndez-Lagares G., Tang Y., Elsheikh M.M., Thompson G.R., Hartigan-O’Connor D.J., Margolis D.M. (2018). HIV latency is reversed by ACSS2-driven histone crotonylation. J. Clin. Investig..

[84] Chávez L., Kauder S., Verdin E. (2011). In vivo, in vitro, and in silico analysis of methylation of the HIV-1 provirus. Methods.

[85] Bednarik D.P., Mosca J.D., Raj N.B. (1987). Methylation as a modulator of expression of human immunodeficiency virus. J. Virol..

[86] Bednarik D.P., Cook J.A., Pitha P.M. (1990). Inactivation of the HIV LTR by DNA CpG methylation: Evidence for a role in latency. EMBO J..

[87] Blazkova J., Trejbalova K., Gondois-Rey F., Halfon P., Philibert P., Guiguen A., Verdin E., Olive D., Van Lint C., Hejnar J. (2009). CpG methylation controls reactivation of HIV from latency. PLoS Pathog..

[88] Kauder S.E., Bosque A., Lindqvist A., Planelles V., Verdin E. (2009). Epigenetic regulation of HIV-1 latency by cytosine methylation. PLoS Pathog..

[89] Bird A. (2002). DNA methylation patterns and epigenetic memory. Genes Dev..

[90] Goll M.G., Bestor T.H. (2005). Eukaryotic cytosine methyltransferases. Annu. Rev. Biochem..

[91] Lyko F. (2018). The DNA methyltransferase family: A versatile toolkit for epigenetic regulation. Nat. Rev. Genet..

[92] Trejbalová K., Kovářová D., Blažková J., Machala L., Jilich D., Weber J., Kučerová D., Vencálek O., Hirsch I., Hejnar J. (2016). Development of 5’ LTR DNA methylation of latent HIV-1 provirus in cell line models and in long-term-infected individuals. Clin. Epigenetics.

[93] Palacios J.A., Pérez-Piñar T., Toro C., Sanz-Minguela B., Moreno V., Valencia E., Gómez-Hernando C., Rodés B. (2012). Long-term nonprogressor and elite controller patients who control viremia have a higher percentage of methylation in their HIV-1 proviral promoters than aviremic patients receiving highly active antiretroviral therapy. J. Virol..

[94] Ghafouri-Fard S., Mahmud Hussen B., Abak A., Taheri M., Abdulmajid Ayatollahi S. (2022). Emerging role of non-coding RNAs in the course of HIV infection. Int. Immunopharmacol..

[95] Lazar D.C., Morris K.V., Saayman S.M. (2016). The emerging role of long non-coding RNAs in HIV infection. Virus Res..

[96] Schroder A.R., Shinn P., Chen H., Berry C., Ecker J.R., Bushman F. (2002). HIV-1 integration in the human genome favors active genes and local hotspots. Cell.

[97] Lewinski M.K., Bisgrove D., Shinn P., Chen H., Hoffmann C., Hannenhalli S., Verdin E., Berry C.C., Ecker J.R., Bushman F.D. (2005). Genome-wide analysis of chromosomal features repressing human immunodeficiency virus transcription. J. Virol..

[98] Han Y., Lassen K., Monie D., Sedaghat A.R., Shimoji S., Liu X., Pierson T.C., Margolick J.B., Siliciano R.F., Siliciano J.D. (2004). Resting CD4+ T cells from human immunodeficiency virus type 1 (HIV-1)-infected individuals carry integrated HIV-1 genomes within actively transcribed host genes. J. Virol..

[99] Liu H., Dow E.C., Arora R., Kimata J.T., Bull L.M., Arduino R.C., Rice A.P. (2006). Integration of human immunodeficiency virus type 1 in untreated infection occurs preferentially within genes. J. Virol..

[100] Shearwin K.E., Callen B.P., Egan J.B. (2005). Transcriptional interference—A crash course. Trends Genet..

[101] Han Y., Lin Y.B., An W., Xu J., Yang H.C., O’Connell K., Dordai D., Boeke J.D., Siliciano J.D., Siliciano R.F. (2008). Orientation-dependent regulation of integrated HIV-1 expression by host gene transcriptional readthrough. Cell Host Microbe.

[102] Lenasi T., Contreras X., Peterlin B.M. (2008). Transcriptional interference antagonizes proviral gene expression to promote HIV latency. Cell Host Microbe.

[103] Telwatte S., Morón-López S., Aran D., Kim P., Hsieh C., Joshi S., Montano M., Greene W.C., Butte A.J., Wong J.K. (2019). Heterogeneity in HIV and cellular transcription profiles in cell line models of latent and productive infection: Implications for HIV latency. Retrovirology.

[104] Winecoff D. (2019). Examining the Role of Transcriptional Interference in HIV Latency. Bachelor’s Thesis.

[105] Yukl S.A., Kaiser P., Kim P., Telwatte S., Joshi S.K., Vu M., Lampiris H., Wong J.K. (2018). HIV latency in isolated patient CD4(+) T cells may be due to blocks in HIV transcriptional elongation, completion, and splicing. Sci. Transl. Med..

[106] Asamitsu K., Fujinaga K., Okamoto T. (2018). HIV Tat/P-TEFb Interaction: A Potential Target for Novel Anti-HIV Therapies. Molecules.

[107] Coull J.J., Romerio F., Sun J.M., Volker J.L., Galvin K.M., Davie J.R., Shi Y., Hansen U., Margolis D.M. (2000). The human factors YY1 and LSF repress the human immunodeficiency virus type 1 long terminal repeat via recruitment of histone deacetylase 1. J. Virol..

[108] Barton K., Margolis D. (2013). Selective targeting of the repressive transcription factors YY1 and cMyc to disrupt quiescent human immunodeficiency viruses. AIDS Res. Hum. Retrovir..

[109] Coiras M., Lopez-Huertas M.R., Perez-Olmeda M., Alcami J. (2009). Understanding HIV-1 latency provides clues for the eradication of long-term reservoirs. Nat. Rev. Microbiol..

[110] Weil R., Israel A. (2004). T-cell-receptor- and B-cell-receptor-mediated activation of NF-kappaB in lymphocytes. Curr. Opin. Immunol..

[111] Zhong H., May M.J., Jimi E., Ghosh S. (2002). The phosphorylation status of nuclear NF-kappa B determines its association with CBP/p300 or HDAC-1. Mol. Cell..

[112] Garcia-Rodriguez C., Rao A. (1998). Nuclear factor of activated T cells (NFAT)-dependent transactivation regulated by the coactivators p300/CREB-binding protein (CBP). J. Exp. Med..

[113] Cron R.Q., Bartz S.R., Clausell A., Bort S.J., Klebanoff S.J., Lewis D.B. (2000). NFAT1 enhances HIV-1 gene expression in primary human CD4 T cells. Clin. Immunol..

[114] Cary D.C., Fujinaga K., Peterlin B.M. (2016). Molecular mechanisms of HIV latency. J. Clin. Investig..

[115] Zhou Q., Yik J.H. (2006). The Yin and Yang of P-TEFb regulation: Implications for human immunodeficiency virus gene expression and global control of cell growth and differentiation. Microbiol. Mol. Biol. Rev..

[116] Peterlin B.M., Price D.H. (2006). Controlling the elongation phase of transcription with P-TEFb. Mol. Cell.

[117] He N., Zhou Q. (2011). New insights into the control of HIV-1 transcription: When Tat meets the 7SK snRNP and super elongation complex (SEC). J. Neuroimmune Pharmacol..

[118] Jefferys S.R., Burgos S.D., Peterson J.J., Selitsky S.R., Turner A.W., James L.I., Tsai Y.H., Coffey A.R., Margolis D.M., Parker J. (2021). Epigenomic characterization of latent HIV infection identifies latency regulating transcription factors. PLoS Pathog..

[119] Nchioua R., Bosso M., Kmiec D., Kirchhoff F. (2020). Cellular Factors Targeting HIV-1 Transcription and Viral RNA Transcripts. Viruses.

[120] Ammosova T., Berro R., Kashanchi F., Nekhai S. (2005). RNA interference directed to CDK2 inhibits HIV-1 transcription. Virology.

[121] Mousseau G., Valente S. (2012). Strategies to Block HIV Transcription: Focus on Small Molecule Tat Inhibitors. Biology.

[122] Schwartz C., Catez P., Rohr O., Lecestre D., Aunis D., Schaeffer E. (2000). Functional interactions between C/EBP, Sp1, and COUP-TF regulate human immunodeficiency virus type 1 gene transcription in human brain cells. J. Virol..

[123] Carroll-Anzinger D., Kumar A., Adarichev V., Kashanchi F., Al-Harthi L. (2007). Human immunodeficiency virus-restricted replication in astrocytes and the ability of gamma interferon to modulate this restriction are regulated by a downstream effector of the Wnt signaling pathway. J. Virol..

[124] Hotter D., Bosso M., Jonsson K.L., Krapp C., Sturzel C.M., Das A., Littwitz-Salomon E., Berkhout B., Russ A., Wittmann S. (2019). IFI16 Targets the Transcription Factor Sp1 to Suppress HIV-1 Transcription and Latency Reactivation. Cell Host Microbe.

[125] Chen Y., Zhang L., Estaras C., Choi S.H., Moreno L., Karn J., Moresco J.J., Yates J.R., Jones K.A. (2014). A gene-specific role for the Ssu72 RNAPII CTD phosphatase in HIV-1 Tat transactivation. Genes Dev..

[126] Compe E., Egly J.M. (2012). TFIIH: When transcription met DNA repair. Nat. Rev. Mol. Cell Biol..

[127] Besnard E., Hakre S., Kampmann M., Lim H.W., Hosmane N.N., Martin A., Bassik M.C., Verschueren E., Battivelli E., Chan J. (2016). The mTOR Complex Controls HIV Latency. Cell Host Microbe.

[128] Readinger J.A., Schiralli G.M., Jiang J.K., Thomas C.J., August A., Henderson A.J., Schwartzberg P.L. (2008). Selective targeting of ITK blocks multiple steps of HIV replication. Proc. Natl. Acad. Sci USA.

[129] Chen S., Yang X., Cheng W., Ma Y., Shang Y., Cao L., Chen S., Chen Y., Wang M., Guo D. (2017). Immune regulator ABIN1 suppresses HIV-1 transcription by negatively regulating the ubiquitination of Tat. Retrovirology.

[130] Booiman T., Loukachov V.V., van Dort K.A., van ‘t Wout A.B., Kootstra N.A. (2015). DYRK1A Controls HIV-1 Replication at a Transcriptional Level in an NFAT Dependent Manner. PLoS ONE.

[131] Jiang G., Espeseth A., Hazuda D.J., Margolis D.M. (2007). c-Myc and Sp1 contribute to proviral latency by recruiting histone deacetylase 1 to the human immunodeficiency virus type 1 promoter. J. Virol..

[132] Marban C., Suzanne S., Dequiedt F., de Walque S., Redel L., Van Lint C., Aunis D., Rohr O. (2007). Recruitment of chromatin-modifying enzymes by CTIP2 promotes HIV-1 transcriptional silencing. EMBO J..

[133] Li Z., Hajian C., Greene W.C. (2020). Identification of unrecognized host factors promoting HIV-1 latency. PLoS Pathog..

[134] Ajasin D., Eugenin E.A. (2020). HIV-1 Tat: Role in Bystander Toxicity. Front. Cell. Infect. Microbiol..

[135] Debaisieux S., Rayne F., Yezid H., Beaumelle B. (2012). The ins and outs of HIV-1 Tat. Traffic.

[136] Rice A.P. (2017). The HIV-1 Tat Protein: Mechanism of Action and Target for HIV-1 Cure Strategies. Curr. Pharm. Des..

[137] Jin H., Li D., Lin M.H., Li L., Harrich D. (2020). Tat-Based Therapies as an Adjuvant for an HIV-1 Functional Cure. Viruses.

[138] Jean M.J., Power D., Kong W., Huang H., Santoso N., Zhu J. (2017). Identification of HIV-1 Tat-Associated Proteins Contributing to HIV-1 Transcription and Latency. Viruses.

[139] Jager S., Cimermancic P., Gulbahce N., Johnson J.R., McGovern K.E., Clarke S.C., Shales M., Mercenne G., Pache L., Li K. (2011). Global landscape of HIV-human protein complexes. Nature.

[140] Dingwall C., Ernberg I., Gait M.J., Green S.M., Heaphy S., Karn J., Lowe A.D., Singh M., Skinner M.A., Valerio R. (1989). Human immunodeficiency virus 1 tat protein binds trans-activation-responsive region (TAR) RNA in vitro. Proc. Natl. Acad. Sci. USA.

[141] Spector C., Mele A.R., Wigdahl B., Nonnemacher M.R. (2019). Genetic variation and function of the HIV-1 Tat protein. Med. Microbiol. Immunol..

[142] Tyagi M., Rusnati M., Presta M., Giacca M. (2001). Internalization of HIV-1 tat requires cell surface heparan sulfate proteoglycans. J. Biol. Chem..

[143] Ruiz A.P., Ajasin D.O., Ramasamy S., DesMarais V., Eugenin E.A., Prasad V.R. (2019). A Naturally Occurring Polymorphism in the HIV-1 Tat Basic Domain Inhibits Uptake by Bystander Cells and Leads to Reduced Neuroinflammation. Sci. Rep..

[144] Mediouni S., Marcondes M.C., Miller C., McLaughlin J.P., Valente S.T. (2015). The cross-talk of HIV-1 Tat and methamphetamine in HIV-associated neurocognitive disorders. Front. Microbiol..

[145] King J.E., Eugenin E.A., Buckner C.M., Berman J.W. (2006). HIV tat and neurotoxicity. Microbes Infect..

[146] Loret E. (2015). HIV extracellular Tat: Myth or reality?. Curr. HIV Res..

[147] Aguilera L.U., Rodriguez-Gonzalez J. (2019). Modeling the effect of tat inhibitors on HIV latency. J. Theor. Biol..

[148] Mousseau G., Mediouni S., Valente S.T. (2015). Targeting HIV transcription: The quest for a functional cure. Curr. Top. Microbiol. Immunol..

[149] Mediouni S., Jablonski J., Paris J.J., Clementz M.A., Thenin-Houssier S., McLaughlin J.P., Valente S.T. (2015). Didehydro-cortistatin A inhibits HIV-1 Tat mediated neuroinflammation and prevents potentiation of cocaine reward in Tat transgenic mice. Curr. HIV Res..

[150] Mediouni S., Chinthalapudi K., Ekka M.K., Usui I., Jablonski J.A., Clementz M.A., Mousseau G., Nowak J., Macherla V.R., Beverage J.N. (2019). Didehydro-Cortistatin A Inhibits HIV-1 by Specifically Binding to the Unstructured Basic Region of Tat. mBio.

[151] Kalantari P., Narayan V., Henderson A.J., Prabhu K.S. (2009). 15-Deoxy-Delta12,14-prostaglandin J2 inhibits HIV-1 transactivating protein, Tat, through covalent modification. FASEB J..

[152] Narayanan A., Sampey G., Van Duyne R., Guendel I., Kehn-Hall K., Roman J., Currer R., Galons H., Oumata N., Joseph B. (2012). Use of ATP analogs to inhibit HIV-1 transcription. Virology.

[153] Wan Z., Chen X. (2014). Triptolide inhibits human immunodeficiency virus type 1 replication by promoting proteasomal degradation of Tat protein. Retrovirology.

[154] Yang M. (2005). Discoveries of Tat-TAR interaction inhibitors for HIV-1. Curr. Drug Targets-Infect. Disord..

[155] Sagnier S., Daussy C.F., Borel S., Robert-Hebmann V., Faure M., Blanchet F.P., Beaumelle B., Biard-Piechaczyk M., Espert L. (2015). Autophagy restricts HIV-1 infection by selectively degrading Tat in CD4+ T lymphocytes. J. Virol..

[156] Lata S., Ali A., Sood V., Raja R., Banerjea A.C. (2015). HIV-1 Rev downregulates Tat expression and viral replication via modulation of NAD(P)H:quinine oxidoreductase 1 (NQO1). Nat. Commun..

[157] Ali A., Banerjea A.C. (2016). Curcumin inhibits HIV-1 by promoting Tat protein degradation. Sci. Rep..

[158] Zhang L., Qin J., Li Y., Wang J., He Q., Zhou J., Liu M., Li D. (2014). Modulation of the stability and activities of HIV-1 Tat by its ubiquitination and carboxyl-terminal region. Cell Biosci..

[159] Gargano B., Fiorillo M., Amente S., Majello B., Lania L. (2008). p14ARF is capable of promoting HIV-1 tat degradation. Cell Cycle.

[160] Ali A., Farooqui S.R., Banerjea A.C. (2019). The host cell ubiquitin ligase protein CHIP is a potent suppressor of HIV-1 replication. J. Biol. Chem..

[161] Li J., Chen C., Ma X., Geng G., Liu B., Zhang Y., Zhang S., Zhong F., Liu C., Yin Y. (2016). Long noncoding RNA NRON contributes to HIV-1 latency by specifically inducing tat protein degradation. Nat. Commun..

[162] Jadlowsky J.K., Nojima M., Schulte A., Geyer M., Okamoto T., Fujinaga K. (2008). Dominant negative mutant cyclin T1 proteins inhibit HIV transcription by specifically degrading Tat. Retrovirology.

[163] Gao W., Li G., Zhao S., Wang H., Huan C., Zheng B., Jiang C., Zhang W. (2021). Deubiquitinating enzyme USP21 inhibits HIV-1 replication by downregulating Tat expression. J. Virol..

[164] Hong H.W., Lee S.W., Myung H. (2013). Induced degradation of Tat by nucleocapsid (NC) via the proteasome pathway and its effect on HIV transcription. Viruses.

[165] Ali A., Raja R., Farooqui S.R., Ahmad S., Banerjea A.C. (2017). USP7 deubiquitinase controls HIV-1 production by stabilizing Tat protein. Biochem. J..

[166] Sivakumaran H., van der Horst A., Fulcher A.J., Apolloni A., Lin M.H., Jans D.A., Harrich D. (2009). Arginine methylation increases the stability of human immunodeficiency virus type 1 Tat. J. Virol..

[167] Mousseau G., Kessing C.F., Fromentin R., Trautmann L., Chomont N., Valente S.T. (2015). The Tat Inhibitor Didehydro-Cortistatin A Prevents HIV-1 Reactivation from Latency. mBio.

[168] Mousseau G., Aneja R., Clementz M.A., Mediouni S., Lima N.S., Haregot A., Kessing C.F., Jablonski J.A., Thenin-Houssier S., Nagarsheth N. (2019). Resistance to the Tat Inhibitor Didehydro-Cortistatin A Is Mediated by Heightened Basal HIV-1 Transcription. mBio.

[169] Kotoku N., Sumii Y., Kobayashi M. (2011). Stereoselective synthesis of core structure of cortistatin A. Org. Lett..

[170] Nicolaou K.C., Sun Y.P., Peng X.S., Polet D., Chen D.Y. (2008). Total synthesis of (+)-cortistatin A. Angew. Chem..

[171] Shi J., Manolikakes G., Yeh C.H., Guerrero C.A., Shenvi R.A., Shigehisa H., Baran P.S. (2011). Scalable synthesis of cortistatin A and related structures. J. Am. Chem. Soc..

[172] Simmons E.M., Hardin-Narayan A.R., Guo X., Sarpong R. (2010). Formal total synthesis of (+/−)-cortistatin A. Tetrahedron.

[173] Liu Q. (2011). Triptolide and its expanding multiple pharmacological functions. Int. Immunopharmacol..

[174] Gao J., Zhang Y., Liu X., Wu X., Huang L., Gao W. (2021). Triptolide: Pharmacological spectrum, biosynthesis, chemical synthesis and derivatives. Theranostics.

[175] Qiu D., Zhao G., Aoki Y., Shi L., Uyei A., Nazarian S., Ng J.C., Kao P.N. (1999). Immunosuppressant PG490 (triptolide) inhibits T-cell interleukin-2 expression at the level of purine-box/nuclear factor of activated T-cells and NF-kappaB transcriptional activation. J. Biol. Chem..

[176] Vispe S., DeVries L., Creancier L., Besse J., Breand S., Hobson D.J., Svejstrup J.Q., Annereau J.P., Cussac D., Dumontet C. (2009). Triptolide is an inhibitor of RNA polymerase I and II-dependent transcription leading predominantly to down-regulation of short-lived mRNA. Mol. Cancer Ther..

[177] Wang Y., Lu J.J., He L., Yu Q. (2011). Triptolide (TPL) inhibits global transcription by inducing proteasome-dependent degradation of RNA polymerase II (Pol II). PLoS ONE.

[178] Liang X., Xie R., Su J., Ye B., Wei S., Liang Z., Bai R., Chen Z., Li Z., Gao X. (2019). Inhibition of RNA polymerase III transcription by Triptolide attenuates colorectal tumorigenesis. J. Exp. Clin. Cancer Res..

[179] Titov D.V., Gilman B., He Q.L., Bhat S., Low W.K., Dang Y., Smeaton M., Demain A.L., Miller P.S., Kugel J.F. (2011). XPB, a subunit of TFIIH, is a target of the natural product triptolide. Nat. Chem. Biol..

[180] Davidson A., Leeper T.C., Athanassiou Z., Patora-Komisarska K., Karn J., Robinson J.A., Varani G. (2009). Simultaneous recognition of HIV-1 TAR RNA bulge and loop sequences by cyclic peptide mimics of Tat protein. Proc. Natl. Acad. Sci. USA.

[181] Murchie A.I., Davis B., Isel C., Afshar M., Drysdale M.J., Bower J., Potter A.J., Starkey I.D., Swarbrick T.M., Mirza S. (2004). Structure-based drug design targeting an inactive RNA conformation: Exploiting the flexibility of HIV-1 TAR RNA. J. Mol. Biol..

[182] Richter S., Parolin C., Gatto B., Del Vecchio C., Brocca-Cofano E., Fravolini A., Palu G., Palumbo M. (2004). Inhibition of human immunodeficiency virus type 1 tat-trans-activation-responsive region interaction by an antiviral quinolone derivative. Antimicrob. Agents Chemother..

[183] Stelzer A.C., Frank A.T., Kratz J.D., Swanson M.D., Gonzalez-Hernandez M.J., Lee J., Andricioaei I., Markovitz D.M., Al-Hashimi H.M. (2011). Discovery of selective bioactive small molecules by targeting an RNA dynamic ensemble. Nat. Chem. Biol..

[184] Davidson A., Begley D.W., Lau C., Varani G. (2011). A small-molecule probe induces a conformation in HIV TAR RNA capable of binding drug-like fragments. J. Mol. Biol..

[185] Abulwerdi F.A., Le Grice S.F.J. (2017). Recent Advances in Targeting the HIV-1 Tat/TAR Complex. Curr. Pharm. Des..

[186] Chiu Y.L., Cao H., Jacque J.M., Stevenson M., Rana T.M. (2004). Inhibition of human immunodeficiency virus type 1 replication by RNA interference directed against human transcription elongation factor P-TEFb (CDK9/CyclinT1). J. Virol..

[187] Gu J., Babayeva N.D., Suwa Y., Baranovskiy A.G., Price D.H., Tahirov T.H. (2014). Crystal structure of HIV-1 Tat complexed with human P-TEFb and AFF4. Cell Cycle.

[188] Tahirov T.H., Babayeva N.D., Varzavand K., Cooper J.J., Sedore S.C., Price D.H. (2010). Crystal structure of HIV-1 Tat complexed with human P-TEFb. Nature.

[189] Fujinaga K. (2020). P-TEFb as A Promising Therapeutic Target. Molecules.

[190] Ali A., Ghosh A., Nathans R.S., Sharova N., O’Brien S., Cao H., Stevenson M., Rana T.M. (2009). Identification of flavopiridol analogues that selectively inhibit positive transcription elongation factor (P-TEFb) and block HIV-1 replication. Chembiochem.

[191] Baumli S., Lolli G., Lowe E.D., Troiani S., Rusconi L., Bullock A.N., Debreczeni J.E., Knapp S., Johnson L.N. (2008). The structure of P-TEFb (CDK9/cyclin T1), its complex with flavopiridol and regulation by phosphorylation. EMBO J..

[192] Chao S.H., Fujinaga K., Marion J.E., Taube R., Sausville E.A., Senderowicz A.M., Peterlin B.M., Price D.H. (2000). Flavopiridol inhibits P-TEFb and blocks HIV-1 replication. J. Biol. Chem..

[193] Chao S.H., Price D.H. (2001). Flavopiridol inactivates P-TEFb and blocks most RNA polymerase II transcription in vivo. J. Biol. Chem..

[194] Agbottah E., de La Fuente C., Nekhai S., Barnett A., Gianella-Borradori A., Pumfery A., Kashanchi F. (2005). Antiviral activity of CYC202 in HIV-1-infected cells. J. Biol. Chem..

[195] Bai J., Sui J., Zhu R.Y., Tallarico A.S., Gennari F., Zhang D., Marasco W.A. (2003). Inhibition of Tat-mediated transactivation and HIV-1 replication by human anti-hCyclinT1 intrabodies. J. Biol. Chem..

[196] Hamasaki T., Okamoto M., Baba M. (2013). Identification of novel inhibitors of human immunodeficiency virus type 1 replication by in silico screening targeting cyclin T1/Tat interaction. Antimicrob. Agents Chemother..

[197] Heredia A., Davis C., Bamba D., Le N., Gwarzo M.Y., Sadowska M., Gallo R.C., Redfield R.R. (2005). Indirubin-3’-monoxime, a derivative of a Chinese antileukemia medicine, inhibits P-TEFb function and HIV-1 replication. Aids.

[198] Sung T.L., Rice A.P. (2009). miR-198 inhibits HIV-1 gene expression and replication in monocytes and its mechanism of action appears to involve repression of cyclin T1. PLoS Pathog..

[199] Van Duyne R., Guendel I., Jaworski E., Sampey G., Klase Z., Chen H., Zeng C., Kovalskyy D., El Kouni M.H., Lepene B. (2013). Effect of mimetic CDK9 inhibitors on HIV-1-activated transcription. J. Mol. Biol..

[200] Pande V., Ramos M.J. (2003). Nuclear factor kappa B: A potential target for anti-HIV chemotherapy. Curr. Med. Chem..

[201] Jean M.J., Fiches G., Hayashi T., Zhu J. (2019). Current Strategies for Elimination of HIV-1 Latent Reservoirs Using Chemical Compounds Targeting Host and Viral Factors. AIDS Res. Hum. Retrovir..

[202] Gupta S.C., Sundaram C., Reuter S., Aggarwal B.B. (2010). Inhibiting NF-kappaB activation by small molecules as a therapeutic strategy. Biochim. Biophys. Acta.

[203] Baba M. (2006). Recent status of HIV-1 gene expression inhibitors. Antiviral. Res..

[204] Victoriano A.F., Okamoto T. (2012). Transcriptional control of HIV replication by multiple modulators and their implication for a novel antiviral therapy. AIDS Res. Hum. Retrovir..

[205] Hiscott J., Kwon H., Génin P. (2001). Hostile takeovers: Viral appropriation of the NF-kappaB pathway. J. Clin. Investig..

[206] Sun S.C. (2017). The non-canonical NF-κB pathway in immunity and inflammation. Nat. Rev. Immunol..

[207] Beinke S., Ley S.C. (2004). Functions of NF-kappaB1 and NF-kappaB2 in immune cell biology. Biochem J..

[208] Karin M., Yamamoto Y., Wang Q.M. (2004). The IKK NF-kappa B system: A treasure trove for drug development. Nat. Rev. Drug Discov..

[209] Ishii Y., Waxman S., Germain D. (2007). Targeting the ubiquitin-proteasome pathway in cancer therapy. Anticancer Agents Med. Chem..

[210] Swinney D.C., Xu Y.Z., Scarafia L.E., Lee I., Mak A.Y., Gan Q.F., Ramesha C.S., Mulkins M.A., Dunn J., So O.Y. (2002). A small molecule ubiquitination inhibitor blocks NF-kappa B-dependent cytokine expression in cells and rats. J. Biol. Chem..

[211] Lin Y.Z., Yao S.Y., Veach R.A., Torgerson T.R., Hawiger J. (1995). Inhibition of nuclear translocation of transcription factor NF-kappa B by a synthetic peptide containing a cell membrane-permeable motif and nuclear localization sequence. J. Biol. Chem..

[212] Choi K.C., Lee Y.H., Jung M.G., Kwon S.H., Kim M.J., Jun W.J., Lee J., Lee J.M., Yoon H.G. (2009). Gallic acid suppresses lipopolysaccharide-induced nuclear factor-kappaB signaling by preventing RelA acetylation in A549 lung cancer cells. Mol. Cancer Res..

[213] Sung B., Pandey M.K., Ahn K.S., Yi T., Chaturvedi M.M., Liu M., Aggarwal B.B. (2008). Anacardic acid (6-nonadecyl salicylic acid), an inhibitor of histone acetyltransferase, suppresses expression of nuclear factor-kappaB-regulated gene products involved in cell survival, proliferation, invasion, and inflammation through inhibition of the inhibitory subunit of nuclear factor-kappaBalpha kinase, leading to potentiation of apoptosis. Blood.

[214] Zhang S., Won Y.K., Ong C.N., Shen H.M. (2005). Anti-cancer potential of sesquiterpene lactones: Bioactivity and molecular mechanisms. Curr. Med. Chem. Anticancer Agents.

[215] Khaled A.R., Butfiloski E.J., Sobel E.S., Schiffenbauer J. (1998). Use of phosphorothioate-modified oligodeoxynucleotides to inhibit NF-kappaB expression and lymphocyte function. Clin. Immunol. Immunopathol..

[216] Bubici C., Papa S., Dean K., Franzoso G. (2006). Mutual cross-talk between reactive oxygen species and nuclear factor-kappa B: Molecular basis and biological significance. Oncogene.

[217] Mihm S., Ennen J., Pessara U., Kurth R., Droge W. (1991). Inhibition of HIV-1 replication and NF-kappa B activity by cysteine and cysteine derivatives. AIDS.

[218] Staal F.J., Roederer M., Herzenberg L.A., Herzenberg L.A. (1990). Intracellular thiols regulate activation of nuclear factor kappa B and transcription of human immunodeficiency virus. Proc. Natl. Acad. Sci. USA.

[219] Schulze-Osthoff K., Beyaert R., Vandevoorde V., Haegeman G., Fiers W. (1993). Depletion of the mitochondrial electron transport abrogates the cytotoxic and gene-inductive effects of TNF. EMBO J..

[220] Manna S.K., Zhang H.J., Yan T., Oberley L.W., Aggarwal B.B. (1998). Overexpression of manganese superoxide dismutase suppresses tumor necrosis factor-induced apoptosis and activation of nuclear transcription factor-kappaB and activated protein-1. J. Biol. Chem..

[221] Makio H., Hiroshi M., Isao S., Masatoshi K., Hirofumi T., Hideyo Y., Michael K., Kiyomi K. (2003). Evidence that reactive oxygen species do not mediate NF-kappaB activation. EMBO J..

[222] Yan C., Dodd T., He Y., Tainer J.A., Tsutakawa S.E., Ivanov I. (2019). Transcription preinitiation complex structure and dynamics provide insight into genetic diseases. Nat. Struct. Mol. Biol..

[223] Davidson L., Muniz L., West S. (2014). 3’ end formation of pre-mRNA and phosphorylation of Ser2 on the RNA polymerase II CTD are reciprocally coupled in human cells. Genes Dev..

[224] Carpenter M.A., Kemp M.G. (2021). Topical treatment of human skin and cultured keratinocytes with high-dose spironolactone reduces XPB expression and induces toxicity. JID Innov..

[225] Shahar O.D., Kalousi A., Eini L., Fisher B., Weiss A., Darr J., Mazina O., Bramson S., Kupiec M., Eden A. (2014). A high-throughput chemical screen with FDA approved drugs reveals that the antihypertensive drug Spironolactone impairs cancer cell survival by inhibiting homology directed repair. Nucleic Acids Res..

[226] Verma D., Thompson J., Swaminathan S. (2016). Spironolactone blocks Epstein-Barr virus production by inhibiting EBV SM protein function. Proc. Natl. Acad. Sci. USA.

[227] Gabbard R.D., Hoopes R.R., Kemp M.G. (2020). Spironolactone and XPB: An Old Drug with a New Molecular Target. Biomolecules.

[228] Lacombe B., Morel M., Margottin-Goguet F., Ramirez B.C. (2016). Specific Inhibition of HIV Infection by the Action of Spironolactone in T Cells. J. Virol..

[229] Formosa T., Winston F. (2020). The role of FACT in managing chromatin: Disruption, assembly, or repair?. Nucleic Acids Res..

